# Screening and Characterization of Phenolic Compounds and Their Antioxidant Capacity in Different Fruit Peels

**DOI:** 10.3390/foods9091206

**Published:** 2020-09-01

**Authors:** Hafiz A. R. Suleria, Colin J. Barrow, Frank R. Dunshea

**Affiliations:** 1 School of Agriculture and Food, Faculty of Veterinary and Agricultural Sciences, The University of Melbourne, Parkville, VIC 3010, Australia; fdunshea@unimelb.edu.au; 2 Centre for Chemistry and Biotechnology, School of Life and Environmental Sciences, Deakin University, Waurn Ponds, VIC 3217, Australia; colin.barrow@deakin.edu.au; 3 Faculty of Biological Sciences, The University of Leeds, Leeds LS2 9JT, UK

**Keywords:** fruit peels, polyphenols, phenolic acids, flavonoids, flavan-3-ols, hydrolysable and condensed tannins, antioxidant activities, LC-MS and HPLC

## Abstract

Fruit peels have a diverse range of phytochemicals including carotenoids, vitamins, dietary fibres, and phenolic compounds, some with remarkable antioxidant properties. Nevertheless, the comprehensive screening and characterization of the complex array of phenolic compounds in different fruit peels is limited. This study aimed to determine the polyphenol content and their antioxidant potential in twenty different fruit peel samples in an ethanolic extraction, including their comprehensive characterization and quantification using the LC-MS/MS and HPLC. The obtained results showed that the mango peel exhibited the highest phenolic content for TPC (27.51 ± 0.63 mg GAE/g) and TFC (1.75 ± 0.08 mg QE/g), while the TTC (9.01 ± 0.20 mg CE/g) was slightly higher in the avocado peel than mango peel (8.99 ± 0.13 mg CE/g). In terms of antioxidant potential, the grapefruit peel had the highest radical scavenging capacities for the DPPH (9.17 ± 0.19 mg AAE/g), ABTS (10.79 ± 0.56 mg AAE/g), ferric reducing capacity in FRAP (9.22 ± 0.25 mg AA/g), and total antioxidant capacity, TAC (8.77 ± 0.34 mg AAE/g) compared to other fruit peel samples. The application of LC-ESI-QTOF-MS/MS tentatively identified and characterized a total of 176 phenolics, including phenolic acids (49), flavonoids (86), lignans (11), stilbene (5) and other polyphenols (25) in all twenty peel samples. From HPLC-PDA quantification, the mango peel sample showed significantly higher phenolic content, particularly for phenolic acids (gallic acid, 14.5 ± 0.4 mg/g) and flavonoids (quercetin, 11.9 ± 0.4 mg/g), as compared to other fruit peel samples. These results highlight the importance of fruit peels as a potential source of polyphenols. This study provides supportive information for the utilization of different phenolic rich fruit peels as ingredients in food, feed, and nutraceutical products.

## 1. Introduction

Food processing industries discard huge amounts of fruit wastes, particularly peels, seeds, and some other fruit residues [1]. These fruit wastes have different challenges for many countries, including Australia. Inappropriate landfill management results in emissions of gases including methane and carbon dioxide, while incomplete incineration involves the subsequent formation of secondary wastes such as dioxins, furans, acid gases, and releases of other dangerous pollutants that can cause serious environmental and health issues [2]. For these reasons, there is an urgent need to find uses for these food wastes, including fruit peel wastes. Some fruit peels have been recycled into products ranging from agricultural compost, biofuel, and citric acid [3]. However, fruit peels also provide an excellent source of carbohydrates, fibre, proteins, and phytochemicals, particularly phenolic compounds with high antioxidant capacities [4]. These components are not generally recovered from peels and so provide a future source of valuable antioxidant ingredients. Polyphenols are a large group of secondary metabolites commonly present in fruits and vegetables, which play a prominent role in human health and nutrition [5]. Phenolic compounds consist of aromatic rings with hydroxyl groups, organic acids, and acylated sugars. These phenolic moieties have high antioxidant activity which prevents the formation of free radicals [6]. The most abundant polyphenols in different fruit peels include flavan-3-ols, flavonols, phenolic acids, anthocyanins, and hydroquinones [7].

The fruit juice industries generate substantial quantities of peel residues during juice processing [8]. The major phenolic compounds present in different fruit peels (apple, pomegranates, mango, pineapple, and citrus peels) include hydroxybenzoic and hydroxycinnamic acids (caffeic acid, gallic acid, protocatechuic acid, and chlorogenic acid), hydrolysable tannins (pedunculagin, punicalin, punicalagin, and ellagic and gallic acids) and flavonoids including anthocyanins [9]. The phenolic compounds identified in avocado and custard apple peels include high contents of condensed tannins and flavonoids including procyanidins [10], whereas those in banana peels are mainly gallocatechin, catechin, and epicatechin [11]. *Prunus* cultivars such as nectarine, peaches, and apricot peels are rich in hydroxycinnamates and flavan-3-ols that have potential antioxidant activities [12]. The antioxidant potential of polyphenols can be estimated with different in vitro spectrophotometric-based methods, including (i) the determination of total phenolics, (ii) free radical scavenging methods, (iii) non-radical redox potential-based methods, and (iv) metal-chelating methods [13]. In addition, polyphenols can also inactivate the Fenton reaction by reacting with different metal ions [14]. For this reason, a set of different in vitro spectrophotometric-based assays with different mechanisms, including total phenolic content (TPC), total flavonoid content (TFC), total tannin content (TTC), 2, 2′-diphenyl-1-picrylhydrazyl (DPPH) free radical scavenging assay, ferric reducing assay (FRAP), 2, 2′-azinobis-(3-ethylbenzothiazoline-6-sulfonic acid) (ABTS) assay and total antioxidant capacity (TAC), were used to estimate overall phenolic contents and map their antioxidant potential [15].

In recent years, there is increasing interest in the extraction of phenolic compounds from different plant materials. Extraction, identification, and characterization of novel phenolics from different plant-based materials are challenging due to their chemical and structural diversity and complexity. The liquid chromatography coupled with electrospray-ionization and quadrupole time-of-flight mass spectrometry (LC-ESI-QTOF-MS/MS) is an innovative tool with high sensitivity and is the most effective method for the characterization of both low and high molecular weight phenolic and non-phenolic compounds [16]. Also, high-performance liquid chromatography (HPLC) coupled with the photodiode array detector (PDA) is a useful tool for quantifying targeted polyphenols. Although several studies have quantified selected phenolic compounds from a range of different fruit by-products using conventional HPLC-UV-based techniques, there is limited literature available on the relative abundance and distribution of numerous phenolic compounds in Australia’s grown fruit peels, particularly using advanced LC-MS/MS characterization methods. As far we know, only some selected phenolic compounds have been characterized in fruit peels using LC-MS/MS [17]. Therefore, extraction, identification, and characterization of phenolics from different fruit peels using advanced analytical techniques including the LC-ESI-QTOF-MS/MS will provide further information in developing innovative functional foods, nutraceuticals, and pharmaceuticals on a commercial scale from these food wastes.

The objective of this study is to determine the phenolic content including TPC, TFC, TTC in twenty (20) different fruit peel samples and assess their antioxidant potential by determining DPPH, TAC, FRAP, and ABTS. Moreover, the identification and characterization of untargeted phenolic compounds were achieved through the LC-ESI-QTOF-MS/MS followed by the quantification of twenty targeted phenolics through HPLC-PDA. This study provides supportive information for the use of different phenolic rich fruit peels as ingredients in food, feed, and nutraceutical products.

## 2. Materials and Methods

### 2.1. Chemicals

In this study, most of the chemicals, reagents, and standards were analytical grade and purchased from Sigma-Aldrich (Castle Hill, NSW, Australia). Gallic acid, L-ascorbic acid, vanillin, hexahydrate aluminium chloride, Folin-Ciocalteu’s phenol reagent, sodium phosphate, iron(III) chloride hexahydrate (Fe[III]Cl_3_.6H_2_O), hydrated sodium acetate, hydrochloric acid, sodium carbonate anhydrous, ammonium molybdate, quercetin, catechin, 2,2′-diphenyl-1-picrylhy-drazyl (DPPH), 2,4,6tripyridyl-s-triazine (TPTZ), and 2,2′-azinobis-(3-ethylbenzothiazoline-6-sulfonic acid) (ABTS) were purchased from the Sigma-Aldrich (Castle Hill, NSW, Australia) for the estimation of polyphenols and antioxidant potential. Sulfuric acid (H_2_SO_4_) with 98% purity was purchased from RCI Labscan (Rongmuang, Thailand). HPLC standards including gallic acid, *p*-hydroxybenzoic acid, caftaric acid, caffeic acid, protocatechuic acid, sinapinic acid, chlorogenic acid, syringic acid, ferulic acid, coumaric acid, catechin, quercetin, quercetin-3-galactoside, diosmin, quercetin-3-glucuronide, epicatechin gallate, quercetin-3-glucoside, kaempferol and kaempferol-3-glucoside were produced by Sigma-Aldrich (Castle Hill, NSW, Australia) for quantification proposes. HPLC and LC-MS grade reagents including methanol, ethanol, acetonitrile, formic acid, and glacial acetic acid were purchased from Thermo Fisher Scientific Inc. (Scoresby, VIC, Australia). To perform various in vitro bioactivities and antioxidant assays, 96 well-plates were bought from the Thermo Fisher Scientific (VIC, Australia). Additionally, HPLC vials (1 mL) were procured from the Agilent technologies (VIC, Australia).

### 2.2. Sample Preparation

**Figure 1:**
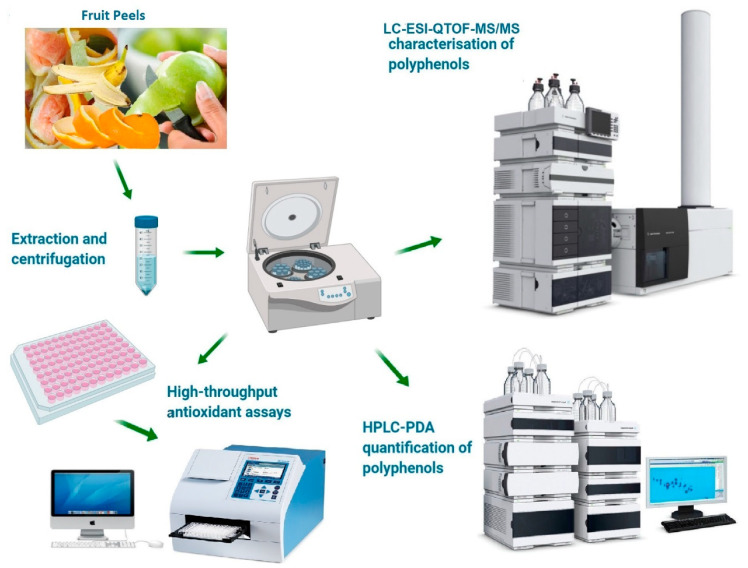
Graphical overview and schematic layout of the proposed research study.

Twenty different Australian grown fresh and mature fruits varieties (2–3 kg) including apple (Royal gala), apricot (Mystery), avocado (Hass), banana (Cavendish), custard apple (African Pride), dragon fruit (Red-fleshed), grapefruit (Thompson), kiwifruit (Hayward), mango (Kensington Pride), lime (Tahitian), melon (Rock melons), nectarine (Fantasia), orange (Navels), papaya (Sunrise Solo), passionfruit (Misty Gem), peach (Florda gold), pear (Packham’s Triumph), pineapple (Aussie Rough), plum (Angeleno), and pomegranate (Griffith) were purchased from a local produce market in Melbourne, Australia. The fruits were manually cleaned, and peels were removed and freeze-dried according to the method of Peng, et al. [18], described in the supplementary material. Figure 1 represents the graphical and schematic layout of our study.

### 2.3. Extraction of Phenolic Compounds

To extract the phenolic compounds, 2.0 ± 0.5 g of each fruit peel powder was mixed with 20 mL 70% ethanol by modifying the method of Gu, et al. [19], explained in the supplementary material.

### 2.4. Estimation of Phenolics and Antioxidant Potential

For the phenolic estimation in selected fruit peel samples, TPC, TFC, and TTC assays were performed, while for measuring their antioxidant capacities, four different types of antioxidant assays including FRAP, DPPH, ABTS, and TAC were performed by adopting our previously published methods of Tang, et al. [20], explained in the supplementary material. The data was determined using a Multiskan^®^ Go microplate photometer (Thermo Fisher Scientific, Waltham, MA, USA).

### 2.5. Characterization and Quantification of Phenolics Using LC-ESI-QTOF-MS/MS and HPLC-PDA

The phenolic compound characterization was performed on an Agilent 1200 HPLC with 6520 Accurate-Mass Q-TOF-MS (Agilent Technologies, Santa Clara, CA, USA). The separation and characterization of phenolics were conducted by adopting our previously published method of Zhong, et al. [21], elaborated in the supplementary material. However, for the quantification of targeted phenolics present in different fruit peel samples was achieved with an Agilent 1200 HPLC coupled with a photodiode array (PDA) detector by following the protocol of Ma, et al. [22], explained in the supplementary material.

### 2.6. Statistical Analysis

All analyses were performed in triplicate, and the results are presented as mean ± standard deviation (*n* = 3). The mean differences between different samples were analyzed by one-way analysis of variance (ANOVA) and Tukey’s honestly significant differences (HSD) multiple rank test at *p* ≤ 0.05. ANOVA was carried out via Minitab 19.0 (Minitab, LLC, State College, PA, USA) and GraphPad Prism 7.05 Software for Windows (GraphPad 7.05 Software, San Diego, CA, USA, www.graphpad.com). For correlations between polyphenol content and antioxidant activities, Pearson’s correlation coefficient at *p* ≤ 0.05 and multivariate statistical analysis including a principal component analysis (PCA), XLSTAT-2019.1.3 were used by Addinsoft Inc. New York, NY, USA.

## 3. Results and Discussion

This study involved the screening and characterization of phenolic compounds with antioxidant potential from twenty different fruit peel samples. An untargeted polyphenol identification and characterization were achieved by the LC-ESI-QTOF-MS/MS, an advanced analytical technique which can provide comprehensive phytochemical screening and MS/MS characterization. For the quantification of phenolic compounds, the twenty most abundant phenolic compounds including (10) phenolic acids and (10) flavonoids present in different fruit peels were targeted and quantified by the HPLC-PDA. A strong correlation between phenolic compound levels and antioxidant activities was observed in all selected fruit peel samples.

### 3.1. Phenolic Estimation (TPC, TFC and TTC)

Fruit peels contain high concentrations of phenolic compounds including flavonoids, phenolic acids, and tannins. The phenolic contents in different fruit peel samples were determined with TPC, TFC, and TTC assays.

Table 1 summarizes the polyphenol concentrations and antioxidant potentials of twenty selected fruit peel samples. The TPC values of these fruit peel samples varied widely, with mango, grapefruit, and lime peel samples exhibiting the highest TPC values (27.51 ± 0.63, 27.22 ± 1.00 and 23.32 ± 2.07 mg GAE/g, respectively), followed by orange and avocado peel samples. The lowest phenolic contents were detected in dragon fruit, nectarine, and passion fruit peels. Comparing all the peel samples, the mango peel sample had significantly higher phenolic contents (*p* < 0.05) than any other fruit peels. Previously, Nguyen, et al. [23] reported significantly higher phenolic contents in mango peels as compared to other tropical fruits, including passion fruit and dragon fruit, which is consistent with our results. In our study, total phenolic content was measured using the Folin-Ciocalteu reagent that has the ability to react with both phenolics and non-phenolic compounds such as ascorbic acid and other reducing substances [24]. Grape and lime peel were reported to be rich in ascorbic acid, which may be one of the contributors to their high total polyphenol content [25]. However, grapefruit and lime peel were previously found to be abundant in polymethoxylated flavones, phenolic acids, and flavanones including naringin and neohesperidin [26]. Previously, similar trends but with higher TPC values were detected in different fruit juices, including grapefruit (657.65 ± 69.20 mg GAE/g), lime (579.41 ± 91.14 mg GAE/g) and orange (523.44 ± 87.20 mg GAE/g) [27]. Nurliyana, et al. [28] also found that dragon peel has a high phenolic content, most likely due to the abundance of betacyanins (pigments) rather than polyphenols, which increased the TPC values [29]. Considering these facts, polyphenol characterization through advanced analytical techniques including LC-MS/MS can provide more reliable and useful information for their applications in different food, feed, nutraceutical, and pharmaceutical industries.

Flavonoids are the predominant class of phenolic substances found in almost all plants, which was determined via the aluminium chloride colorimetric method in this study. Aluminium chloride reacts with carbonyl group present in flavonoids, forming a stable complex [30]. The highest amount of flavonoid was found in the mango peel (1.75 ± 0.08 mg QE/g), followed by pineapple and banana peels (1.47 ± 0.07 and 1.32 ± 0.12 mg QE/g, respectively). Marina and Noriham [31] also reported higher flavonoid contents in mango peel than other tropical fruit peels such as papaya and guava peels, which is consistent with our study. Previously, Morais, et al. [32] determined the TFC in different parts of the avocado (*Persea americana*), and found that avocado peels had more flavonoids than seeds and pulp. Ayala-Zavala, et al. [33] also reported that the peels of tropical exotic fruits like avocado, pineapple, banana, papaya, passion fruit, and melon contain more phenolic acids and flavonoids than pulp. Overall, TFC values of our twenty different fruit peels were slightly higher than previously reported values, which may be due to the difference in the growing area, climatic conditions, varietal differences, and extraction. Fruits growing under different climatic regions have different flavonoid content in their peels, the peels being the outer part of fruit bodies exposed to more to sunlight as compared to pulp, leading to the synthesis of the abundant and diverse nature of flavonoids. Nogata, et al. [34] reported that the flavonoid contents in the outer layer of citrus fruits are higher than the inner layers and pulps. The flavonoid profile differs among species and cultivars of the same fruits grown in different regions under different climatic conditions, soil characteristics, and cultivation techniques [35]. Moreover, the efficiency of the extraction of flavonoids also varies under different extraction conditions, such as the type of solvents, solvent concentration, extraction time and temperature, solvent-to-solid ratio, etc. [36,37].

Tannins are also one of the important groups of phenolic compounds which can be classified into hydrolysable tannins and condensed tannins. Avocado peels exhibited the highest TTC values of 9.01 ± 0.20 mg CE/g, followed by mango (8.99 ± 0.13 mg CE/g), lime, and custard apple peels (8.42 ± 0.63 and 8.32 ± 0.56 mg CE/g, respectively), while few tannins were detected in dragon fruit, melon, nectarine, passion fruit, peach and pear peels. Overall, most of our TTC values are in accordance with previously published work, while we also had high values of tannins in mango peel as compared to the previously published literature. Previously, the mango fruit peel has already been reported to be a rich source of hydrolysable tannins, while hydrolysable tannins can decrease significantly during the ripening process [38]. One of the possible reasons might be the difference in sample preparations, storage conditions, and extraction techniques. In our study, all the fruit peels were freeze-dried prior to the extraction of polyphenols; it has been reported that freeze-drying facilitates the overall polyphenol extraction. Freeze-drying can also preserve the highest percentage of condensed tannins as compared to other conventional drying methods. Freeze-drying also helps to accelerate the release of bounded phenolic compound [39], deactivating oxidative and hydrolytic enzymes, improving the extraction and protecting the phenolic compounds [40].

### 3.2. Antioxidant Potential (DPPH, ABTS, FRAP and TAC)

To further investigate the antioxidant potential of the twenty different fruit peels, different antioxidant assays based on different mechanisms were applied in this study. Antioxidant assays including DPPH and ABTS were used to measure the radical scavenging ability, while FRAP and TAC assays were used to determine the reducing power of samples. The results shown in Table 1 were reported in mg ascorbic acid equivalents (AAE) per g of samples (mg AAE/g).

The DPPH assay is widely used to determine the free radical scavenging activity, which is mainly attributed to polyphenols [15]. Grapefruit, mango, and avocado peels exhibit higher DPPH radical scavenging ability (9.17 ± 0.19, 8.67 ± 0.49 and 8.67 ± 0.44 mg AAE/g, respectively). Previously, different varieties of mango peel extracts have shown concentration-dependent DPPH free radical scavenging activity [41]. Most of our DDPH values are in accordance with the previously published literature. Moreover, the DPPH assay showed significantly higher levels of antioxidant capacity in freeze-dried fruit peels as compared to fresh fruit peels. The freeze-drying process generates redox-active metabolites that can scavenge and neutralize free radicals [42]. The DPPH assay is one of the non-specific free radical scavenging assays since it measures scavenged free radicals from both phenolic and non-phenolic compounds, including ascorbic acid. Therefore, the antioxidant potential of plant polyphenols cannot be properly assessed only through DPPH assays. For this reason, a set of different in vitro reagent-based assays can be applied to estimate antioxidant potential, while the confirmation of these antioxidant compounds can be achieved through the LC-MS characterization.

The ABTS assay is another widely used method for determining the antiradical scavenging abilities based on the hydrogen atom donating tendency of phenolic compounds. The scavenged ABTS free radicals were measured using a colorimetric assay where antioxidants in samples reduce ABTS^+^ and form a stable free radical [15]. The ABTS assay exhibits high similarity with that of the DPPH assay with the highest ABTS value from the grapefruit peel with 10.79 ± 0.56 mg AAE/g, followed by the mango peel (9.32 ± 0.24 mg AAE/g), kiwi fruit peel (8.95 ± 0.18 mg AAE/g), and avocado peel (7.19 ± 0.72 mg AAE/g) samples. In comparison, banana, dragon fruit, melon, nectarine, passion fruit, peach, pear, and plum peels exhibited relatively low ABTS radical scavenging ability. Previously, a similar ABTS^∙+^scavenging tendency was found in white and pink freeze-dried grapefruit peel extracts [42]. Pal, et al. [43] also found the ABTS radical scavenging ability in kiwi fruit at different ripening stages. Tremocoldi, et al. [44] reported slightly higher ABTS radical scavenging activities in different avocado varieties, including Hass and Fuerte peel samples, as compared to our results. Ortega-Arellano, et al. [45] also reported the ABTS antioxidant activity for both Hass and Reed peels, which is consistent with our results.

The FRAP assay evaluates the ability of samples to donate electrons to reduce a Fe^+3^-TPTZ complex to a blue Fe^+2^-TPTZ complex. Grapefruit peel exhibited the highest FRAP reducing power with 9.22 ± 0.25 mg AAE/g, followed by mango peel (6.19 ± 0.26 mg AAE/g), avocado peel (3.65 ± 0.07 mg AAE/g) and apple peel samples (3.20 ± 0.04 mg AAE/g), while the FRAP reducing power from dragon fruit, melon, passion fruit, pear, and plum peels were relatively low as compared to other fruit peels. Previously, Oboh and Ademosun [46] also reported high FRAP activity in orange and apple peels that was attributed to their bound phenolics compounds and flavonoids. Furthermore, FRAP activities previously reported in other fruit peels including kiwifruit, lime, pineapple, banana, and mango was also in accordance with our study [47].

The total antioxidant capacity (TAC) assay is based on an electron transfer mechanism. This assay is very similar to FRAP, where molybdenum (VI) will be reduced to molybdenum (V) through antioxidant compounds or phenolic compounds. Similar to the results of FRAP assay, the highest TAC values were reported in the grapefruit peel (8.77 ± 0.34 mg AAE/g), followed by mango, avocado, and apple peels (6.19 ± 0.23, 4.50 ± 0.16 and 2.97 ± 0.16 mg AAE/g, respectively). In comparison, dragon fruit, kiwi fruit, melon, nectarine, papaya, peach, pear, and plum peels had relatively low TAC values. The strong antioxidant activities including DPPH, ABTS, and FRAP of different citrus fruits have already been reported, while grapefruit had the strongest antioxidant potential [48]. Antioxidant assays involved multiple reactions and mechanisms to estimate the antioxidant potential of any plant material, and unfortunately, there is no single method that can accurately reflect the overall antioxidant potential due to the complex nature of phytochemicals. For this reason, the MS/MS characterization is one of the key areas in phytochemical research to used compute overall phenolic compounds and their antioxidant potential.

In general, grapefruit, mango, and avocado peels exhibit distinctive antioxidant activity in four different types of antioxidant assays. Our polyphenolic and antioxidant results indicated that further research is needed to determine the actual contribution of polyphenols toward the antioxidant potential by minimizing other distracting factors of in vitro reagent-based assays, including the contribution of non-phenolic compounds toward the antioxidant potential.

### 3.3. LC-ESI-QTOF-MS/MS Characterization

LC-MS/MS has been widely used for the identification and characterization of bioactive compounds, including phenolics from different fruits, vegetable, and medicinal plants. An untargeted qualitative analysis of phenolics from twenty different fruit peel samples was achieved via LC-ESI-QTOF-MS/MS analysis in both negative and positive modes of ionization (Table S1, Figures S1 and S2—Supplementary Data). Phenolics present in different fruit peel samples were tentatively identified and characterized from their *m/z* value and MS spectra in both negative and positive modes of ionization ([M − H]^−^/[M + H]^+^) using Agilent LC-MS Qualitative Software and Personal Compound Database and Library (PCDL). Compounds with mass error < ± 5 ppm and PCDL library score more than 80 were selected for further MS/MS identification and *m/z* characterization and verification purposes. In our study, LC-MS/MS enabled the tentative identification and characterization of 176 phenolics in twenty different fruit peel samples, including phenolic acids (49), flavonoids (86), lignans (11), stilbene (5) and other polyphenols (25) listed in Table S1 (Supplementary data).

#### 3.3.1. Phenolic Acids

Phenolic acids are the most abundant bioactive compounds present in different fruits [5]. In our study, a total of 49 phenolic acids were tentatively characterized, including hydroxybenzoic acids (12), hydroxycinnamic acids (31), hydroxyphenylacetic acids (2), and hydroxyphenylpropanoic acids (4).

Hydroxybenzoic acids are widely present in different fruits such as mango, apple, custard apple, citrus, strawberries, and raspberries with significant antioxidant potential. Compound **1** presenting in mango, pear and kiwifruit was proposed as vanillic acid 4-sulfate based on the observed *m*/*z* at 246.9911 in negative ionization mode and further confirmed by the MS/MS experiment which displayed a characteristic loss of SO_3_ (80 Da) at *m/z* 167 [49]. Most of the phenolic acids showed the loss of CO_2_ (44 Da) and hexosyl moiety (162 Da) [50]. Compound **3** (*m*/*z* 169.0146), compound **6** (*m*/*z* 137.0244) and compound **8** (*m*/*z* 153.0193) were identified as gallic acid, 2-hydroxybenzoic acid and 2,3-dihydroxybenzoic acid, showing product ions at *m*/*z* 125, at *m*/*z* 93 and at *m*/*z* 109, represented the loss of CO_2_ from the precursor ions [50,51]. Previously, Kim, et al. [52] had also tentatively identified gallic acid from white and red dragon fruit peel and pulp samples.

Hydroxycinnamic acids contained collectively a larger number of detected compounds than in any other subclass in this study. In our study, a total of 31 hydroxycinnamic acids were identified with remarkable antioxidant potential. Six caffeic acid derivatives were successfully identified in our work. Compound **15** (*m*/*z* 341.0861) and compound **26** (*m*/*z* 355.0686) exhibited a product ion at *m*/*z* 179 (caffeic acid ion) by losing glucoside (162 Da) and glucuronide (176 Da) in negative mode and identified as caffeoyl glucose and caffeic acid 3*-O-*glucuronide [53].

**Figure 2:**
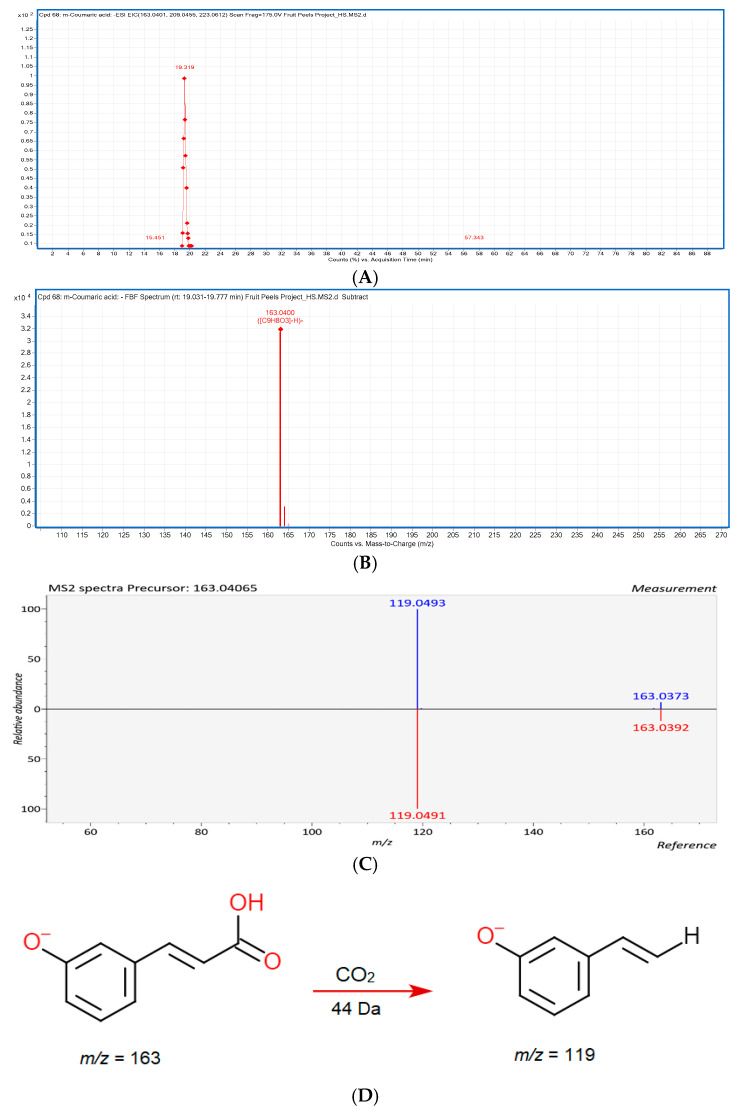
The LC-ESI-QTOF-MS/MS characterization of *m*-coumaric acid. (**A**) A chromatograph of *m*-coumaric acid (Compound 25, Table S1—Supplementary Data) in the negative mode [M − H]^−^ which was tentatively identified and characterized in fifteen different fruit peel samples; (**B**) a mass spectrum of *m*-coumaric acid with a precursor of *m*/*z* 163.0406 in the apple peel; (**C**) MS/MS spectrum of *m*-coumaric acid with the product ion of *m/z* 119 (confirmed from online LC-MS library and database); (**D**) a fragmentation pattern of the *m*-coumaric acid in negative mode [M − H]^−^, with precursor of *m/z* 163 and a product ion of *m/z* 119 due to the loss of CO_2_.

Ferulic acid (Compound **23**) was also observed in eight different peel samples. In an MS^2^ experiment, ferulic acid displayed the product ions at *m*/*z* 178, *m*/*z* 149, and *m*/*z* 134, indicating the loss of CH_3_, CO_2_, and CH_3_ with CO_2_ from the precursor, respectively [54]. Compound **25** (RT = 19.319 min) was tentatively identified as *m*-coumaric acid with the precursor [M − H]^−^ *m*/*z* at 163.0406 and confirmed by the MS/MS spectra (Figure 2), which exhibited the fragments at *m*/*z* 119 due to the loss of CO_2_ [54]. Compound **47** (dihydroferulic acid 4*-O-*glucuronide, *m*/*z* at 371.0986) and compound **49** (dihydrocaffeic acid 3*-O-*glucuronide, *m*/*z* at 357.0811) were both detected only in the negative ionization mode, and the characteristic loss of the glucuronide (176 Da) moiety was observed in both compounds, which produced the fragment ions at *m*/*z* 195 and at *m*/*z* 181, respectively [55].

#### 3.3.2. Flavonoids

Flavonoids (in total 86) are the most abundant class with antioxidant potential found in the fruit peels. Flavonoids were divided into eight subclasses, including flavanols (11), flavones (12), flavanones (8), flavonols (19), dihydrochalcones (3), dihydroflavonols (2), anthocyanins (12) and Isoflavonoids (19).

A total of eight flavanones was discovered in the peels. Quercetin 3’*-O-*glucuronide (Compound **82**) and myricetin 3*-O-*arabinoside (Compound **83**) were found in both modes and tentatively identified by the precursor ions [M − H]^−^ *m/z* at 477.067 and [M − H]^−^ *m/z* at 449.0716. The product ion at *m/z* 301 in the MS^2^ spectrum of quercetin 3’*-O-*glucuronide was produced by the loss of glucuronide (176 Da) from the precursor [56], and the peaks at *m/z* 317 (loss of pentose moiety, 132 Da) confirmed the identity of myricetin 3*-O-*arabinoside [57].

#### 3.3.3. Other Polyphenols

A total of 25 other polyphenols were identified from the peels, which were further divided into hydroxycoumarins (5), hydroxybenzaldehydes (2), hydroxybenzoketones (2), hydroxyphenylpropenes (1), curcuminoids (3), furanocoumarins (1), phenolic terpenes (2), tyrosols (5) and other polyphenols (4).

Coumarin (Compound **138**) and scopoletin (Compound **139**) were found in both negative and positive modes and tentatively identified according to the precursors [M + H]^+^ at *m/z* 147.0448 and [M − H]^−^ at *m/z* 191.0345. In the MS^2^ experiment of 147.0448, peaks at *m/z* 103 [M + H − CO_2_] and *m/z* 91 [M + H − 2CO] achieved the identification of coumarin, and in the MS/MS spectra of *m/z* 191.0345, peaks at 176 [M − H − 15, loss of CH_3_] are characteristic for scopoletin [58,59].

#### 3.3.4. Lignans

A total of eleven lignans were identified in most of the fruit peels. Compounds **161** and **163** presenting in the positive mode were identified as enterolactone and schisandrin C according to the *m/z* 299.1283 and *m/z* 385.1652, respectively. The MS/MS experiment achieved the identification of these lignans. Enterolactone exhibited the fragment ions at *m/z* 281, 187, and 165, representing the loss of H_2_O, C_6_H_8_O_2_ and C_9_H_8_O_2_, respectively [60]. The presence of schisantherin C was verified by the product ions at *m/z* 370 (loss of CH_3_, 15 Da), *m/z* 315 (loss of C_5_H_10_, 70 Da) and *m/z* 300 (loss of CH_3_ and C_5_H_10_, 85 Da) [61].

#### 3.3.5. Stilbenes

A total of five stilbenes were identified in different fruit peel samples. Resveratrol (Compound **173,** [M − H]^−^ *m/z* at 227.0709 presenting in custard apple and avocado peels) and resveratrol 5-*O*-glucoside (Compound **174**, [M − H]^−^ *m/z* at 389.1245 appearing in passion fruit, pomegranate, and kiwi fruit peels) were detected in both ionization modes. In the MS^2^ spectra, Resveratrol showed the characteristic *m/z* at 212 (loss of CH_3_), 185 (loss of CHCOH), 157 (loss of CHCOH and CO), and 143 (loss of CHCOH and C_2_H_2_O) [62]. The excepted loss of glucoside (162 Da) was observed in the MS^2^ fragmentation of resveratrol 5-*O*-glucoside, which allowed the identification of this compound [63].

The LC-MS/MS characterizations of phenolic compounds presented in different fruit peels have remarkable antioxidant capacities. Most of the hydroxycinnamic hydroxybenzoic acids and their derivatives and flavonoid and their derivatives have strong free radical scavenging ability. The presence of these phenolics in different fruit peel samples indicates that these food wastes could be valuable sources of natural antioxidant compounds. In short, these fruit peels could be utilized in different food, feed, nutraceutical, and pharmaceutical industries.

### 3.4. Distribution of Phenolic Compounds—Venn Diagram

**Figure 3:**
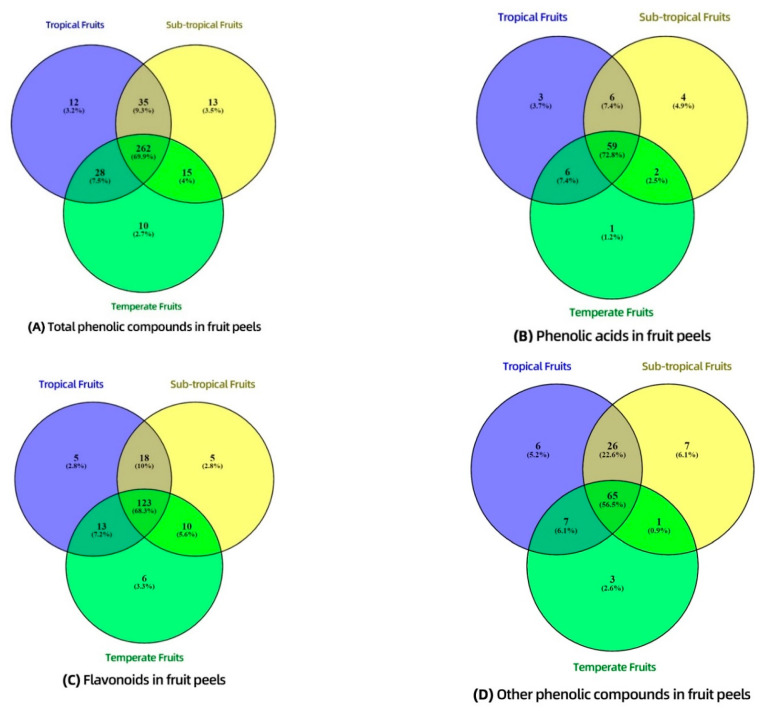
Venn diagram of phenolic compounds presented in different fruit peel samples grown in different regions. (**A**) shows the relations of total phenolic compounds present in different fruit peel samples grown in three different zones. (**B**) shows the relations of phenolic acids present in different fruit peel samples. (**C**) shows the relations of flavonoids present in different fruit peel samples. (**D**) shows the relations of other phenolic compounds present in different fruit peel samples.

To further investigate the distribution of phenolic compounds in different fruit peels, Venn diagrams were generated among fruits grown in different climate zones including tropical, sub-tropical, and temperate (Figure 3). Although the aim of this study was not to explore the relationship between growing regions and phenolic contents in different fruit peel samples, we tentatively characterized their phenolic profiling through Venn diagrams. This preliminary analysis indicates that it is worth further exploring the relationship between growing regions and phenolic contents in different fruit peel samples. The comparison showed that there are differences in the phenolic compositions of fruits grown in different climate zones, and so it may be possible to optimize phenolic levels in these fruits and their peels through the targeted selection of the growing location.

Fruit peel samples were divided into three groups according to their growing regions, which were tropical (banana, custard apple, dragon fruit, mango, papaya, and pineapple peels), sub-tropical (pomegranate, passion fruit, orange, grapefruit, avocado, lime peels), and temperate (apple, apricot, kiwi fruit, peach, pear, melon, plum, nectarine peels).

From Figure 3A, a total of 375 phenolics were tentatively identified in all twenty selected fruit peels. Among “*total phenolic compounds*”, 69.9% of them were commonly identified in all three zones, including tropical, sub-tropical, and temperate regions. From Figure 3B,C, a total of 72.8% of the “*phenolic acids*” and 68.3% of the “*flavonoids*” were commonly identified in all three zones. The proportions of common phenolic acids and flavonoids shared by all the fruit peels were almost similar to that of total phenolic compounds, which indicated the compositions of these compounds were similar in tropical, sub-tropical, and temperate fruits, despite different growing regions. However, Figure 3D shows that “*other phenolic compounds*” had only 56.5% of commonly identified compounds in the three groups, the proportions of which were much lower than those in the total phenolic compounds. The lower proportion of shared compounds of *“other phenolic compound”* indicated that they might be the main contributors responsible for the differences in overall phenolic concentrations and antioxidant activities of different fruit peels collected from three different climatic zones. Additionally, tropical and sub-tropical fruits were more similar in the compositions of other phenolic compounds, while temperate fruits had a quite different composition. The difference may be explained by a previous study, which indicated that tropical fruits often had richer phenolic contents and stronger antioxidant capabilities than temperate fruits due to the presence of some phenolic compounds in tropical fruits that functioned as lipid peroxidation inhibitors and decreased deleterious effects in plants caused by the strong ultraviolet radiation in tropical regions [64]. For example, stilbenes possess an antioxidant ability that can decrease oxidative stress caused by UV irradiation, as well as for the defense system of plants against fungi and bacteria [65]. Also, other phenolic compounds with anti-insect functions might exist exclusively in tropical fruits, as a warmer climate usually favors pest threats [66,67].

In this work we found that there is a strong relationship between growing regions and phenolic contents in different fruit peel samples, and we elucidate the differences in the compositions of phenolic compounds, particularly “*other phenolic compounds*”. Further work is required to explore the impacts of individual phenolics.

### 3.5. HPLC-PDA Quantitative Analysis

HPLC has been widely used as an effective tool for the identification and quantification of phenolic compounds in different fruit and vegetable samples. The twenty most abundant phenolic compounds present in the different fruit peels, including 10 phenolic acids and 10 flavonoids, were selected for quantification. Tables 2 and 3 show the quantified phenolic acids and flavonoids by comparing retention time with reference standards, and results were calculated using standard curves.

#### 3.5.1. Phenolic Acids

In our study, ten targeted phenolic acids were quantified in the twenty fruit peels. Table 2 showed that the mango peel was most abundant in terms of the overall phenolic acids (72.2 ± 4.5 mg/g) and most of the individual phenolic acid, while melon peels significantly had the lowest overall phenolic acid content. Mango peels significantly had the highest phenolic content for seven out of ten targeted phenolic acids, including gallic acid (14.5 ± 0.4 mg/g), chlorogenic acid (13.8 ± 0.9 mg/g), caffeic acid (4.5 ± 0.4 mg/g), *p*-hydroxybenzoic acid (10.5 ± 0.4 mg/g), syringic acid (11.5 ± 0.7 mg/g), ferulic acid (6.3 ± 0.4 mg/g), and coumaric acid (5.1 ± 0.2 mg/g), respectively. Previously, Palafox-Carlos, et al. [68] detected gallic acid, chlorogenic acid, and protocatechuic acids in different mango varieties, including in both pulp and peels. Chlorogenic acid was the most abundant in their study, while gallic acid was the most abundant in our study. However, Kim, et al. [69] reported that gallic acid was the predominant phenolic acid in mango peels, which is in agreement with our results. In another study, Hu, et al. [70], reported a gallic acid concentration of 0.08–0.59 mg/g among mango peel samples, which is much lower than our results. These variations can be explained by the variability of phenolic content with cultivar type and maturity, growing regions, and climatic conditions.

Another study of Marina and Noriham [31] indicated that mango peels had higher phenolic content than papaya peels, which also agrees with our quantification results for the ten targeted phenolic acids. Moreover, Gorinstein, et al. [71] also reported a similar trend that mango had significantly higher phenolic contents than avocado peel samples. However, they also reported a higher phenolic content in kiwifruit than in mango, which is in contrast with our results. The difference might be caused by the difference in varieties and growing conditions as they, used grown fruits from Singapore, while our study was conducted on grown fruits from Australia. Apart from mango, other tropical fruits, including banana, custard apple, dragon fruit, papaya, passion fruit, and pineapple peels, did not show significantly higher phenolic contents than other temperate or subtropical fruits, although some of these fruits, such as the banana, were reported to have high phenolic contents and antioxidant ability [72].

Pomegranate was another fruit other than mango which had a significantly higher content for most of the phenolic acids. Previously, Li, et al. [73] detected 249.4 ± 17.2 mg/g phenolic contents in pomegranate peels, which indicated that this fruit was an excellent source of phenolics. Moreover, the study of Marina and Noriham [31] also indicated that pomegranates possessed high phenolic contents. From our results, similar conclusions can be postulated, as pomegranate has a significantly higher phenolic acid content compared with other fruit peels. Additionally, Pal, et al. [74] reported approximately a three-fold higher phenolic content in the pomegranate peel than in the orange peel, which is consistent with our study. Apart from pomegranate, grapefruit and lime peels were also quantified in our study and significantly showed the highest contents for several phenolic acids. Previously, Sir Elkhatim, et al. [75] compared the phenolic contents between peels of citrus fruits including orange, grapefruit, and lime, and reported that grapefruit peels had the highest phenolic content, followed by lime and orange peels, which showed a similar pattern with our results for the targeted phenolic acids. Li, et al. [76] also reported similar results that the grapefruit peel had the highest phenolic contents compared with other citrus fruit peels.

As for temperate fruits, apple peels had significantly higher contents of protocatechuic acid (7.4 ± 0.4 mg/g) than all other fruits. While the apple peel did not have higher overall phenolic acid contents among all the 20 fruits, it is one of the most widely consumed fruits known for its antioxidant ability [77], and importantly, the peel is often consumed. Previously, Russell, et al. [78] reported a higher content of phenolic acids, including gallic acid, protocatechuic acid, *p*-hydroxybenzoic acid, syringic acid, and sinapinic acid, in apple peels than in the peel of pears of Scottish varieties, which is consistent with our results. The study of Mihailović, et al. [79] indicated that chlorogenic acid was the most dominant phenolic acid presented in the apple peel, which is in agreement with our study, which detected the highest chlorogenic acid content of 11.2 ± 0.1 mg/g for apple peels. Moreover, Veberic, et al. [80] also reported that chlorogenic acid was the most abundant phenolic acid in the apple peel with the content range of 4.1–79.5 mg/100 g. The variation can be attributed to a difference in apple varieties. Previous studies have suggested that most tropical fruits have higher phenolic contents than temperate fruits, as phenolic compounds are essential for inhibiting lipid peroxidation and deleterious effects in plant tissues caused by strong ultraviolet radiation in tropical areas [64]. It can also be concluded from previous studies that, although some temperate fruits were already potential phenolic sources, topical fruits had richer phenolic contents, which makes them better sources of phenolic acids [64]. In our study, the tropical fruit mango showed significantly higher phenolic acid content in the peel than all the sub-tropical and temperate fruits, which is consistent with previous studies.

#### 3.5.2. Flavonoids

Flavonoids are the largest group of phenolics and are present in most of the fruits. Among the fruit peels investigated, the mango peel has the highest content for overall flavonoids (57.1 ± 2.4 mg/g), while passion fruit had the lowest (10.4 ± 1.4 mg/g) listed in Table 3.

Mango peels showed similarly high contents for flavonoids as for phenolic acids, significantly with the highest contents of epicatechin gallate (3.2 ± 0.9 mg/g), quercetin-3-galactoside (10.9 ± 0.1 mg/g), quercetin-3-glucuronide (11.5 ± 0.7 mg/g), quercetin (11.9 ± 0.4 mg/g), and kaempferol (9.8 ± 0.7 mg/g). Previously, catechin and quercetin-3-galactoside were quantified by López-Cobo, et al. [81] in different mango peel samples. Compared with other fruits, Marina and Noriham [31] reported higher catechin and epicatechin contents in mango peels than other tropical fruit peels, such as papaya peel and guava peel, which is consistent with our study. However, a few studies reported lower flavonoids in mango pulp as compared to kiwifruit and avocado pulp, which did not agree with our fruit peel extracts [71]. The contradictory results might be explained by previous literature indicating that peels contained more flavonoids as compared to pulp [68].

Apart from mango peel, dragon fruit peel was also found to be abundant with flavonoids while catechin was dominantly detected in it with a concentration of 7.5 ± 0.9 mg/g. Previously, flavonoids including kaempferol and quercetin derivatives were detected and quantified in dragon fruit peels [82]. The pineapple peel sample had a relatively low flavonoid content among all the twenty fruits which showed a different pattern from mango and dragon fruit peels, but these results agree with the previous study of Silva, et al. [83], who reported significantly higher flavonoid contents in mango, papaya, and passion fruit than in pineapple, in which only a few flavonoids were detected in the pineapple peel sample. The pomegranate peel sample also had higher flavonoids (35.7 ± 4.7 mg/g) similar to phenolic contents. For individual flavonoids, pomegranate peel had the highest epicatechin content (4.1 ± 0.3 mg/g). Previously, Li, Guo, Yang, Wei, Xu and Cheng [73] reported higher flavonoids in pomegranate peel than our results, which may be because we only quantified the ten most abundant flavonoids across the fruit samples observed, and there is a chance that individual fruits may have high concentrations of a flavonoid outside this group. Another study showed that flavonoid contents in pomegranate and mango juices were significantly lower than the phenolic acid contents, which is consistent with our results [68]. Our results indicated that both mango and pomegranate peels are excellent sources of phenolic compounds.

For citrus fruit peels, the kaempferol-3-glucoside content was highest in lime peel (3.7 ± 0.4 mg/g), which is higher than those in orange, grapefruit, and pomegranate. Previously, Singh and Immanuel [84] reported similar results that lime peel had a higher total flavonoid content compared with other citrus species, such as orange. However, a more recent study of Sir Elkhatim, Elagib, and Hassan [75] showed that orange peel contained higher amounts of flavonoids than lime and grapefruit peels, which is in contrast with our results. The variation can be attributed to the difference in fruit varieties and extraction methods. In temperate fruits, quercetin-3-glucoside was the most abundant in apple peel, with a concentration of 4.5 ± 0.9 mg/g. Previously, Schieber, et al. [85] also reported quercetin-3-glucoside was present in apple pomace at a low concentration. Another study of Mihailović, Mihailovic, Kreft, Ciric, Joksović and Djurdjevic [79] reported flavonoids including catechin (0.187 ± 0.007 mg/g) and quercitrin (0.256 ± 0.002 mg/g) from peels of wild apple varieties, which were also detected in our study. In summary, all twenty fruit peel samples have a considerable quantity of phenolic compounds, including both phenolic acids & flavonoids, and these fruit peels are potential commercial sources of these phenolics.

### 3.6. Heat Map and Hierarchical Clustering Phenolic Compound Analysis

**Figure 4:**
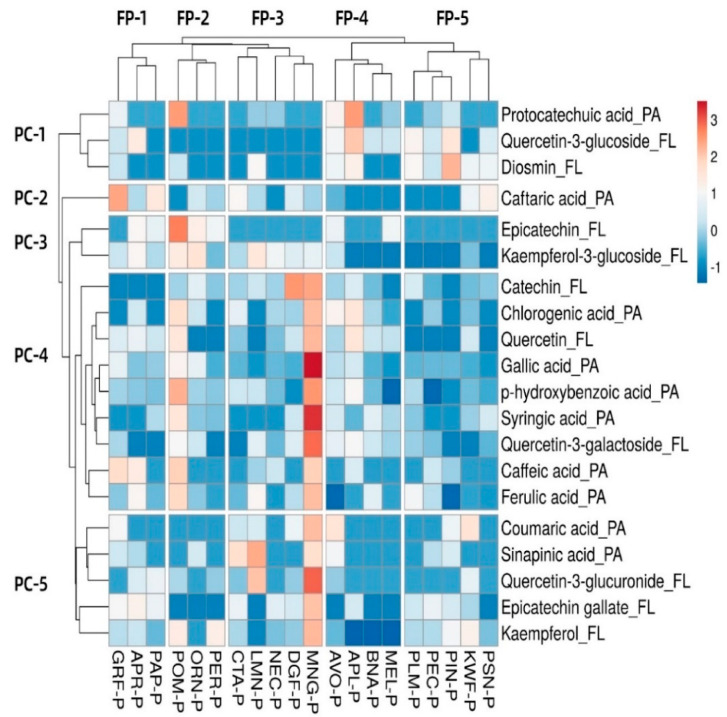
Heatmap showing phenolic compounds’ distribution and concentration among twenty fruit peel samples. Red boxes mean concentrations are higher among different fruit peel samples. Blue boxes mean lower concentrations. PA: phenolic acids; FL: flavonoids; FP 1-5: fruit peel clusters 1; PC 1-5: phenolic compound clusters. Fruit peel samples were mentioned in abbreviations. Apple peel “APL-P”, Apricot peel “APR-P”, Avocado peel “AVO-P”, Banana peel “BNA-P”, Custard apple peel “CTA-P”, Dragon fruit peel “DGF-P”, Grapefruit peel “GRF-P”, kiwifruit peel “KWF-P”, Lime peel “LMN-P”, Mango peel “MNG-P”, Melon peel “MEL-P”, Nectarine peel “NEC-P”, Orange peel xORN-P”, Papaya peel “PAP-P”, Passionfruit peel “PSN-P”, Peach peel “PEC-P”, Pear peel “PER-P”, Pineapple peel “PIN-P”, Plum peel “PLM-P” and Pomegranate peel “POM-P”.

For further analyzing the hierarchical clustering of targeted phenolic compounds in the twenty selected fruit peels, a heat map was constructed (Figure 4). The distance measure used for determining the similarity between fruits and compounds was the correlation, while the clustering method used for rows and columns was based on average concentration. For tree ordering, the tightest clusters were clustered first.

In the heat map, five clusters in rows and columns were generated and highlighted by the hierarchical clustering; different clusters of samples indicate significant differences in phenolic profiles. The color difference showed the abundance of phenolic acids and flavonoids in different fruit peels. From the results, it can be observed that MGN-P, DGF-P, NEC-P, LMN-P, and CTA-P were clustered together in the group (FP-3), which shared similar patterns of phenolic contents. Within this cluster, MGN-P had red color areas for gallic acid and syringic acid, representing higher contents. Previously, Pereira-Netto [64] reported that tropical fruits shared similarly higher contents of phenolics than temperate fruits, which agrees with the clustering, where tropical fruits MGN-P, DGF-P, LMN-P, and CTA-P were grouped together.

Phenolic compounds were also grouped into five main clusters (PC-1, PC-2, PC-3, PC-4, and PC-5) in the dendrogram and were further grouped into different sub-clusters according to the similarity of their concentration patterns in the twenty fruit peel samples. Overall, PC-1 to PC-5 clusters indicated that several phenolic acids (caffeic acid, ferulic acid, coumaric acid, sinapinic acid) and flavonoids (quercectin-3-glucournoide, epicatechin gallate and kaempferol) had greater similarity in terms of the concentration among different fruit peel samples. However, some phenolic acids (caftaric acid, protocatechuic acid) and flavonoids (epicatechin and kaempferol-3-glucoside) showed variability with respect to other phenolic compound clusters.

### 3.7. Correlation between Phenolic Compounds, Targeted Phenolics Quantified through HPLC-PDA and Antioxidant Assays

**Figure 5:**
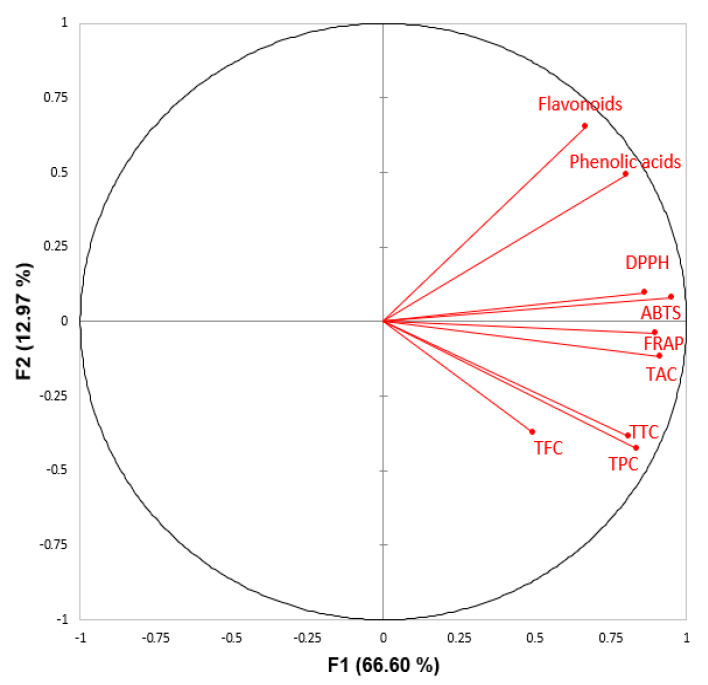
Principal component analysis (PCA) of the phenolic content (TPC, TFC, TTC, phenolic acids and flavonoids—quantified through HPLC-PDA) and antioxidant activities (DPPH, ABTS, FRAP, and TAC) of twenty different fruit peel samples.

The correlation between phenolic content (TPC, TFC, TTC, phenolic acids and flavonoids—quantified through HPLC-PDA) and antioxidant activities (DPPH, FRAP, ABTS, and TAC) was performed with a Pearson’s correlation test (Table 4). In addition, principal components analysis (PCA, Figure 5) was performed to investigate the overall similarities and differences between the phenolic content, targeted phenolic acid, and flavonoids quantified through HPLC in different peels of fruit samples, and the relationship between the various methods used in the evaluation of the antioxidant potential. The targeted (10) phenolic acids and (10) flavonoids were calculated by summarizing the content of the proposed compounds in the HPLC-PDA table to investigate the correlations between overall phenolics and their antioxidant activities.

A total of 79.57% variability of the initial data can be explained by the first two factors (F1 and F2) in Figure 5. Regarding antioxidant assays, DPPH, FRAP, ABTS, and TAC were strongly correlated with each other (*p* ≤ 0.01). This significantly positive correlation was previously reported by Floegel, et al. [86]. They found that both DPPH and ABTS assays evaluate the free radical scavenging ability, and the ABTS assay can better reflect the hydrophilic, lipophilic, and high-pigmented antioxidants in fruits compared to the DPPH assay. The high correlation between DPPH, ABTS, FRAP, and TAC indicated that phenolic compounds present in twenty different fruit peel extracts exhibit the strong scavenging ability of DPPH, ABTS-reducing ability, and ferric ion- and phosphomolybdate ion-reducing abilities, respectively. The significantly positive correlations between FRAP and other antioxidant assays were in agreement with a previous study [87].

The TPC was highly significantly correlated with four antioxidant assays (DPPH, ABTS, FRAP, and TAC), which suggested that phenolic compounds are primary contributors to the antioxidant activities of the twenty different fruit peel samples. These results are in agreement with our previously published studies on phenolic compounds in different fruits and vegetable pulp samples and their antioxidant potential [19]. In addition, TPC were strongly correlated with TTC with *r* = 0.932, *p* ≤ 0.01. However, a non-significant correlation between TFC and antioxidant assays was found, indicating that the contribution from flavonoids to the antioxidant potential of some peel samples was limited. The TFC method used in this study only targeted specific flavonoids, because the aluminum chloride selectively reacts with flavonols and the flavone luteolin [88], which may explain the non-significant correlations. In addition, strong correlations between TTC and four antioxidant assays were found, indicating that tannin present in selected fruit peel samples had a significant contribution to the antioxidant activities.

The phenolic acids content detected in HPLC was highly significantly correlated with most of the antioxidant assays (DPPH, ABTS, FRAP, and TAC) with *r* = 0.761, 0.628, 0.614, 0.640, respectively (*p* ≤ 0.05), indicating that phenolic acids were one of the significant contributors to the antioxidant activities. Flavonoids detected by HPLC were also significantly correlated with most of the antioxidant assays, which was not consistent with the correlation results between the TFC value and antioxidant assays discussed before. One of the reasons might be that we selected only 10 of the most abundant flavonoids across all the fruit peels for quantification purposes, while TFC assays specifically react with all types of flavonoids. In addition, the overall flavonoids detected by HPLC were not correlated with the TFC value (*r* = 0.232), which might be due to the high proportion of other subclasses of flavonoids rather than our targeted (10) flavonoids. Overall, both phenolic acids and flavonoids were strongly correlated with antioxidant assays, which indicated that both phenolic classes have strong antioxidant activities.

## 4. Conclusions

In conclusion, most of the selected fruit peels were found to have considerable amounts of phenolic content with very high in vitro antioxidant potential. The TPC, TFC, DPPH, FRAP, TAC and ABTS scavenging activity was higher in mango peel as compared to other fruit peels. The mango peel sample also showed significantly higher phenolic compounds, including gallic acid and quercetin, as compared to other fruit peel samples. The LC-ESI-QTOF-MS/MS technique was successfully applied for characterization of the phenolic compounds in different fruit peels; a total of 176 phenolic compounds were tentatively characterized. Quantification by HPLC-PDA also verified that fruit peels are rich in phenolic compounds. The obtained results supported the idea that fruit peels are a potential food waste source of phenolic compounds, with high antioxidant potential that has potential utility in food, feed, and nutritional supplements. In the future, in vitro digestibility, bioavailability, bioaccessibility, toxicological, and animal studies are required for developing these different fruit peels as commercial ingredients.

## Figures and Tables

**Table 1:** The polyphenol concentrations and antioxidant potentials of twenty different selected fruit peels.

Sample	TPC (mg GAE/g)	TFC (mg QE/g)	TTC (mg CE/g)	DPPH (mg AAE/g)	ABTS (mg AAE/g)	FRAP (mg AAE/g)	TAC (mg AAE/g)
Apple peel	10.82 ± 0.51 ^e^	1.22 ± 0.10 ^b^^–^^e^	2.25 ± 0.12 ^e^	5.20 ± 0.25 ^b^	4.96 ± 0.17 ^d^	3.20 ± 0.04 ^d^	2.97 ± 0.16 ^d^
Apricot peel	5.60 ± 0.27 ^f,g^	1.22 ± 0.09 ^b^^–^^e^	1.07 ± 0.05 ^f^	3.73 ± 0.55 ^c^	3.21 ± 0.04 ^e^	2.27 ± 0.11 ^e^	2.28 ± 0.04 ^e^^,^^f^
Avocado peel	18.79 ± 1.46 ^c^	1.24 ± 0.11 ^b^^–^^d^	9.01 ± 0.20 ^a^	8.67 ± 0.44 ^a^	7.19 ± 0.72 ^c^	3.65 ± 0.07 ^c^	4.50 ± 0.16 ^c^
Banana peel	6.13 ± 0.25 ^f,g^	1.32 ± 0.12 ^b^^,^^c^	1.22 ± 0.08 ^f^	1.20 ± 0.12 ^e^	1.31 ± 0.03 ^g^^,^^h^	0.81 ± 0.03 ^i^^,^^j^	2.36 ± 0.22 ^e^^,^^f^
Custard apple peel	15.72 ± 0.74 ^d^	1.21 ± 0.08 ^b^^–^^e^	8.32 ± 0.56 ^a^^–^^c^	2.52 ± 0.52 ^d^	4.00 ± 0.44 ^e^	1.51 ± 0.02 ^f^	2.58 ± 0.04 ^d^^,^^e^
Dragon fruit peel	0.45 ± 0.12 ^k^	0.03 ± 0.01 ^h^	0.03 ± 0.01 ^h^	1.03 ± 0.16 ^e^	0.56 ± 0.08 ^h^	0.06 ± 0.01 ^l^	0.19 ± 0.02 ^i^
Grapefruit peel	27.22 ± 1.00 ^a^	0.82 ± 0.14 ^f^	7.60 ± 0.35 ^c^	9.17 ± 0.19 ^a^	10.79 ± 0.56 ^a^	9.22 ± 0.25 ^a^	8.77 ± 0.34 ^a^
kiwi fruit peel	5.30 ± 0.40 ^g,h^	0.45 ± 0.06 ^g^	3.51 ± 0.33 ^d^	5.03 ± 0.39 ^b^	8.95 ± 0.18 ^b^	1.13 ± 0.10 ^g^^–^^i^	0.79 ± 0.05 ^h^
Lime peel	23.32 ± 2.07 ^b^	1.14 ± 0.17 ^c^^–^^e^	8.42 ± 0.63 ^a^^,^^b^	2.73 ± 0.34 ^d^	1.46 ± 0.14 ^g^	0.92 ± 0.07 ^h^^–^^j^	2.27 ± 0.08 ^e^^,^^f^
Mango peel	27.51 ± 0.63 ^a^	1.75 ± 0.08 ^a^	8.99 ± 0.13 ^a^	8.67 ± 0.49 ^a^	9.32 ± 0.24 ^b^	6.19 ± 0.26 ^b^	6.19 ± 0.23 ^b^
Melon peel	2.39 ± 0.02 ^i–k^	0.03 ± 0.01 ^h^	0.02 ± 0.01 ^h^	0.48 ± 0.28 ^e^	1.16 ± 0.20 ^g^^,^^h^	0.08 ± 0.01 ^l^	0.93 ± 0.23 ^g^^–^^h^
Nectarine peel	1.53 ± 0.04 ^j,k^	0.09 ± 0.01 ^h^	0.23 ± 0.18 ^h^	1.29 ± 0.09 ^e^	1.25 ± 0.13 ^g^^,^^h^	0.91 ± 0.07 ^h^^–^^j^	0.97 ± 0.05 ^g^^,^^h^
Orange peel	21.31 ± 1.37 ^b^	1.08 ± 0.06 ^c^^–^^f^	8.12 ± 0.26 ^b^^,^^c^	4.79 ± 0.31 ^b^	3.36 ± 0.16 ^e^	2.44 ± 0.12 ^e^	2.55 ± 0.08 ^d^^,^^e^
Papaya peel	3.13 ± 0.15 ^h–j^	1.06 ± 0.07 ^c^^–^^f^	1.09 ± 0.04 ^f^	1.13 ± 0.11 ^e^	3.30 ± 0.17 ^e^	0.91 ± 0.07 ^i^^,^^j^	1.12 ± 0.13 ^g^^,^^h^
Passion fruit peel	1.55 ± 0.21 ^j,k^	0.04 ± 0.01 ^h^	0.19 ± 0.02 ^h^	0.72 ± 0.13 ^e^	1.04 ± 0.07 ^g^^,^^h^	0.42 ± 0.04 ^k^	1.32 ± 0.05 ^g^
Peach peel	5.84 ± 0.33 ^f,g^	1.02 ± 0.08 ^d^^–^^f^	0.16 ± 0.05 ^h^	1.33 ± 0.11 ^e^	1.03 ± 0.06 ^g^^,^^h^	0.89 ± 0.07 ^i^^,^^j^	1.13 ± 0.07 ^g^^,^^h^
Pear peel	4.30 ± 0.29 ^g–i^	1.07 ± 0.12 ^c^^–^^f^	0.10 ± 0.03 ^h^	0.84 ± 0.12 ^e^	1.21 ± 0.06 ^g^^,^^h^	0.65 ± 0.08 ^j^^,^^k^	1.18 ± 0.03 ^g^^,^^h^
Pineapple peel	7.83 ± 0.35 ^f^	1.47 ± 0.07 ^b^	1.23 ± 0.05 ^f^	1.30 ± 0.07 ^e^	2.36 ± 0.06 ^f^	1.30 ± 0.16 ^f^^,^^g^	2.00 ± 0.14 ^f^
Plum peel	4.81 ± 0.30 ^g,h^	0.96 ± 0.08 ^e^^,^^f^	0.29 ± 0.05 ^g^^,^^h^	1.01 ± 0.10 ^e^	1.19 ± 0.08 ^g^^,^^h^	0.71 ± 0.04 ^j^^,^^k^	0.87 ± 0.04 ^h^
Pomegranate peel	3.89 ± 0.21 ^g–i^	0.97 ± 0.10 ^e^^,^^f^	0.99 ± 0.02 ^f^^–^^g^	4.60 ± 0.08 ^b^^,^^c^	3.34 ± 0.09 ^e^	1.25 ± 0.13 ^f^^–^^h^	2.40 ± 0.18 ^e^^,^^f^

All values are expressed as mg/g mean ± standard deviation (*n* = 3). Alphabetic letters indicate the significant difference (*p* < 0.05) in a row using a one-way analysis of variance (ANOVA) and Tukey’s test. TPC, Total phenolic content; TFC, total flavonoid content; TTC, total tannins content; FRAP, ferric reducing antioxidant power assay; DPPH, 2,2′-diphenyl-1-picrylhydrazyl assay; ABTS, 2,2′-azino-bis-3-ethylbenzothiazoline-6-sulfonic acid assay; TAC, total antioxidant capacity; GAE, gallic acid equivalents; CE, catechin equivalents; QE, quercetin equivalents; AAE, ascorbic acid equivalents.

**Table 2:** Phenolic acids quantified in different fruit peel samples using HPLC-PDA.

Fruit Peels	Gallic Acid	Protocatechuic Acid	Caftaric Acid	Chlorogenic Acid	*p*-hydroxybenzoic Acid	Caffeic Acid	Syringic Acid	Coumaric Acid	Ferulic Acid	Sinapinic Acid	Sum of Phenolic Acids
APL-P	4.2 ± 0.9 ^d^	7.4 ± 0.4 ^a^	-	11.2 ± 0.1 ^b^	6.5 ± 0.8 ^c^	2.1 ± 0.9 ^c^	1.1 ± 0.7 ^g^	-	1.2 ± 0.3 ^h^	-	33.7 ± 1.5 ^C^
APR-P	2.1 ± 0.6 ^g^	-	2.4 ± 0.4 ^e^	5.9 ± 0.2 ^e^	3.1 ± 0.3 ^f^	3.5 ± 0.1 ^b^	-	-	4.5 ± 0.1 ^c^	1.3 ± 0.1 ^e^	22.8 ± 1.7 ^D^
AVO-P	3.2 ± 0.5 ^e^	4.2 ± 0.2 ^b^	1.2 ± 0.1 ^g^	9.5 ± 0.1 ^c^	4.5 ± 0.6 ^d^	-	3.5 ± 0.3 ^d^	4.1 ± 0.1 ^b^	-	2.7 ± 0.6 ^c^	32.9 ± 2.5 ^C^
BNA-P	1.2 ± 0.2 ^i^	-	-	4.5 ± 0.5 ^f^	2.8 ± 0.1 ^g^	-	4.1 ± 0.5 ^c^	-	3.7 ± 0.2 ^d^	-	16.3 ± 1.9 ^G^
CTA-P	1.4 ± 0.2 ^h^	-	4.7 ± 0.9 ^c^	7.5 ± 0.3 ^d^	4.5 ± 0.9 ^d^	-	-	1.8 ± 0.4 ^f^	1.8 ± 0.3 ^g^	3.9 ± 0.8 ^b^	25.6 ± 1.6 ^D^
DGF-P	1.1 ± 0.5 ^i^	-	3.5 ± 0.5 ^d^	4.1 ± 0.9 ^g^	1.2 ± 0.7 ^i^	-	3.1 ± 0.9 ^d^	2.8 ± 0.1 ^d^	2.7 ± 0.8 ^e^	-	18.5 ± 2.1 ^F^
GRF-P	5.4 ± 0.9 ^c^	3.4 ± 0.4 ^c^	7.8 ± 0.5 ^a^	-	3.5 ± 0.9 ^e^	4.2 ± 0.5 ^a^	-	3.1 ± 0.8 ^c^	2.1 ± 0.4 ^f^	1.7 ± 0.7 ^d^	31.2 ± 1.9 ^C^
KWF-P	1.1 ± 0.9 ^i^	-	4.2 ± 0.4 ^c^	3.2 ± 0.5 ^i^	2.8 ± 0.1 ^g^	-	2.1 ± 0.1 ^e^	4.1 ± 0.8 ^b^	1.2 ± 0.7 ^h^	-	18.7 ± 1.7 ^F^
LMN-P	-	1.2 ± 0.8 ^e^	2.4 ± 0.5 ^e^	-	4.2 ± 0.4 ^d^	1.2 ± 0.6 ^e^	-	2.1 ± 0.4 ^e^	4.5 ± 0.9 ^c^	4.9 ± 0.7 ^a^	20.5 ± 2.1 ^E^
MNG-P	14.5 ± 0.4 ^a^	-	2.1 ± 0.1 ^f^	13.8 ± 0.9 ^a^	10.5 ± 0.4 ^a^	4.5 ± 0.4 ^a^	11.5 ± 0.7 ^a^	5.1 ± 0.2 ^a^	6.3 ± 0.4 ^a^	3.9 ± 0.9 ^b^	72.2 ± 4.5 ^A^
MEL-P	-	1.1 ± 0.7 ^e^	-	1.6 ± 0.3 ^j^	-	-	2.3 ± 0.1 ^e^	-	1.2 ± 0.2 ^h^	-	6.2 ± 1.2 ^M^
NEC-P	1.5 ± 0.7 ^h^	1.2 ± 0.2 ^e^	-	4.5 ± 0.4 ^f^	2.8 ± 0.1 ^g^	1.7 ± 0.9 ^d^	-	-	1.1 ± 0.1 ^h^	-	12.8 ± 1.9 ^I^
ORN-P	5.4 ± 0.9 ^c^	-	3.1 ± 0.4 ^d^	5.6 ± 0.3 ^e^	3.6 ± 0.1 ^e^	-	1.8 ± 0.2 ^f^	-	2.1 ± 0.7 ^f^	1.8 ± 0.2 ^d^	23.4 ± 2.3 ^D^
PAP-P	2.4 ± 0.7 ^f^	-	5.6 ± 0.1 ^b^	-	2.9 ± 0.2 ^g^	-	2.4 ± 0.9 ^e^	-	1.8 ± 0.4 ^g^	-	15.1 ± 1.1 ^H^
PSN-P	-	-	5.2 ± 0.8 ^b^	-	2.1 ± 0.4 ^h^	-	3.5 ± 0.3 ^d^	-	1.1 ± 0.1 ^h^	-	11.9 ± 1.9 ^J^
PEC-P	1.5 ± 0.4 ^h^	1.2 ± 0.1 ^e^	-	3.7 ± 0.9 ^h^	-	1.8 ± 0.2 ^d^	-	-	2.7 ± 0.1 ^e^	1.4 ± 0.9 ^e^	12.3 ± 1.3 ^I^
PER-P	1.1 ± 0.7 ^i^	-	2.1 ± 0.8 ^f^	-	3.2 ± 0.3 ^f^	-	1.5 ± 0.4 ^f^	-	1.2 ± 0.1 ^h^	-	9.1 ± 1.7 ^L^
PIN-P	1.5 ± 0.9 ^h^	2.1 ± 0.2 ^d^	-	-	1.2 ± 0.1 ^i^	1.1 ± 0.5 ^e^	-	2.8 ± 0.3 ^d^	-	1.9 ± 0.7 ^d^	10.6 ± 1.9 ^K^
PLM-P	1.4 ± 0.3 ^h^	-	-	-	3.8 ± 0.1 ^e^	-	1.7 ± 0.7 ^f^	-	4.2 ± 0.4 ^c^	-	11.1 ± 2.1 ^J^
POM-P	6.7 ± 0.1 ^b^	7.4 ± 0.6 ^a^	-	11.8 ± 0.7 ^b^	9.8 ± 0.1 ^b^	4.5 ± 0.7 ^a^	6.7 ± 0.9 ^b^	-	5.8 ± 0.2 ^b^	-	52.7 ± 3.9 ^B^

All values are expressed as “mg/g”, mean ± standard deviation (*n* = 3). Alphabetic letters indicate significant difference (*p* < 0.05) in a row using a one-way analysis of variance (ANOVA) and Tukey’s test. Fruit peel samples were mentioned in abbreviations. Apple peel “APL-P”, Apricot peel “APR-P”, Avocado peel “AVO-P”, Banana peel “BNA-P”, Custard apple peel “CTA-P”, Dragon fruit peel “DGF-P”, Grapefruit peel “GRF-P”, Kiwifruit peel “KWF-P”, Lime peel “LMN-P”, Mango peel “MNG-P”, Melon peel “MEL-P”, Nectarine peel “NEC-P”, Orange peel “ORN-P”, Papaya peel “PAP-P”, Passionfruit peel “PSN-P”, Peach peel “PEC-P”, Pear peel “PER-P”, Pineapple peel “PIN-P”, Plum peel “PLM-P”, and Pomegranate peel “POM-P”.

**Table 3:** Flavonoids quantified in different fruit peel samples using HPLC-PDA.

Fruit Peels	Catechin	Epicatechin	Epicatechin Gallate	Quercetin-3-Galactoside	Quercetin-3-Glucuronide	Quercetin-3-Glucoside	Kaempferol-3-Glucoside	Diosmin	Quercetin	Kaempferol	Sum of Flavonoids
APL-P	3.2 ± 0.8 ^b^	-	1.5 ± 0.1 ^d^	5.7 ± 0.6 ^b^	-	4.5 ± 0.9 ^a^	-	2.1 ± 0.4 ^b^	9.6 ± 0.9 ^b^	-	26.6 ± 1.9 ^C^
APR-P	-	2.1 ± 0.8 ^b^	2.3 ± 0.1 ^b^	-	3.8 ± 0.7 ^d^	3.5 ± 0.2 ^b^	3.2 ± 0.4 ^b^	-	6.9 ± 0.7 ^c^	4.5 ± 0.1 ^d^	26.3 ± 2.1 ^C^
AVO-P	2.1 ± 0.9 ^d^	1.8 ± 0.4 ^c^	-	4.9 ± 0.7 ^c^	1.7 ± 0.8 ^f^	2.8 ± 0.1 ^c^	1.9 ± 0.5 ^e^	1.7 ± 0.1 ^c^	3.9 ± 0.9 ^g^	2.9 ± 0.4 ^f^	23.7 ± 2.7 ^D^
BNA-P	1.5 ± 0.7 ^e^	-	-	3.8 ± 0.9 ^d^	-	1.7 ± 0.1 ^e^	-	-	4.8 ± 0.8 ^e^	-	11.8 ± 2.1 ^H^
CTA-P	2.1 ± 0.4 ^d^	-	1.9 ± 0.7 ^c^	-	1.4 ± 0.1 ^f^	-	1.7 ± 0.5 ^e^	-	3.2 ± 0.9 ^h^	3.9 ± 0.4 ^d^	14.2 ± 1.9 ^G^
DGF-P	7.5 ± 0.9 ^a^	-	1.5 ± 0.1 ^d^	4.5 ± 0.7 ^c^	1.7 ± 0.4 ^f^	-	2.4 ± 0.7 ^d^	-	4.9 ± 0.3 ^e^	3.5 ± 0.7 ^e^	26.0 ± 1.1 ^C^
GRF-P	-	-	2.1 ± 0.7 ^b^	2.9 ± 0.1 ^e^	-	1.7 ± 0.7 ^e^	1.9 ± 0.3 ^e^	1.1 ± 0.7 ^d^	5.9 ± 0.1 ^d^	4.2 ± 0.9 ^d^	19.8 ± 1.9 ^E^
KWF-P	1.5 ± 0.2 ^e^	-	1.1 ± 0.9 ^e^	-	4.5 ± 0.3 ^c^	-	1.2 ± 0.8 ^f^	1.7 ± 0.1 ^c^	5.4 ± 0.2 ^d^	7.1 ± 0.7 ^b^	22.5 ± 2.7 ^D^
LMN-P	2.7 ± 0.1 ^c^	-	-	4.8 ± 0.7 ^c^	8.7 ± 0.2 ^b^	-	3.7 ± 0.4 ^a^	1.9 ± 0.6 ^b^	-	1.2 ± 0.1 ^h^	23.0 ± 1.3 ^D^
MNG-P	7.1 ± 0.3 ^a^	-	3.2 ± 0.9 ^a^	10.9 ± 0.1 ^a^	11.5 ± 0.7 ^a^	-	2.7 ± 0.4 ^c^	-	11.9 ± 0.4 ^a^	9.8 ± 0.7 ^a^	57.1 ± 2.4 ^A^
MEL-P	-	1.9 ± 0.3 ^c^	-	2.8 ± 0.9 ^e^	-	1.7 ± 0.1 ^e^	-	-	4.5 ± 0.3 ^f^	-	10.9 ± 1.2 ^I^
NEC-P	2.1 ± 0.8 ^d^	-	1.7 ± 0.1 ^d^	1.8 ± 0.7 ^g^	-	-	2.9 ± 0.7 ^c^	-	2.9 ± 0.9 ^i^	3.1 ± 0.1 ^e^	14.5 ± 2.1 ^G^
ORN-P	3.5 ± 0.1 ^b^	2.4 ± 0.4 ^b^	-	3.8 ± 0.2 ^d^	-	-	3.9 ± 0.1 ^a^	-	-	1.9 ± 0.8 ^g^	15.5 ± 1.7 ^F^
PAP-P	-	1.7 ± 0.3 ^c^	1.9 ± 0.9 ^c^	-	4.7 ± 0.1 ^c^	-	1.7 ± 0.9 ^e^	-	4.9 ± 0.4 ^e^	2.9 ± 0.2 ^f^	17.8 ± 2.1 ^F^
PSN-P	1.8 ± 0.7 ^e^	-	-	1.7 ± 0.1 ^f^	-	2.1 ± 0.9 ^d^	-	1.7 ± 0.1 ^c^	-	3.1 ± 0.8 ^e^	10.4 ± 1.4 ^I^
PEC-P	1.2 ± 0.5 ^f^	-	1.9 ± 0.3 ^c^	2.1 ± 0.4 ^f^	-	1.8 ± 0.2 ^e^	-	1.1 ± 0.1 ^d^	-	4.1 ± 0.7 ^d^	12.2 ± 1.7 ^H^
PER-P	1.8 ± 0.7 ^e^	1.9 ± 0.9 ^c^	-	-	1.7 ± 0.4 ^f^	-	1.1 ± 0.1 ^f^	-	-	7.4 ± 0.7 ^b^	13.9 ± 2.1 ^G^
PIN-P	-	-	1.5 ± 0.1 ^d^	-	-	3.7 ± 0.1 ^b^	-	3.1 ± 0.6 ^a^	-	6.5 ± 0.7 ^c^	14.8 ± 1.9 ^G^
PLM-P	3.1 ± 0.1 ^b^	-	1.4 ± 0.9 ^d^	2.7 ± 0.3 ^e^	-	3.1 ± 0.5 ^c^	-	1.9 ± 0.7 ^b^	-	4.1 ± 0.2 ^d^	16.3 ± 2.1 ^F^
POM-P	2.1 ± 0.1 ^d^	4.1 ± 0.3 ^a^	-	5.7 ± 0.1 ^b^	2.1 ± 0.7 ^e^	-	3.6 ± 0.2 ^a^	1.1 ± 0.3 ^d^	9.4 ± 0.9 ^b^	7.6 ± 0.7 ^b^	35.7 ± 4.7 ^B^

All values are expressed as “mg/g”, mean ± standard deviation (*n* = 3). Alphabetic letters indicate the significant difference (*p* < 0.05) in a row using ANOVA and Tukey’s test. Fruit peel samples were mentioned in abbreviations. Apple peel “APL-P”, Apricot peel “APR-P”, Avocado peel “AVO-P”, Banana peel “BNA-P”, Custard apple peel “CTA-P”, Dragon fruit peel “DGF-P”, Grapefruit peel “GRF-P”, Kiwifruit peel “KWF-P”, Lime peel “LMN-P”, Mango peel “MNG-P”, Melon peel “MEL-P”, Nectarine peel “NEC-P”, Orange peel “ORN-P”, Papaya peel “PAP-P”, Passionfruit peel “PSN-P”, Peach peel “PEC-P”, Pear peel “PER-P”, Pineapple peel “PIN-P”, Plum peel “PLM-P” and Pomegranate peel “POM-P”.

**Table 4:** Pearson’s correlation coefficients (r) between phenolic content (TPC, TFC, TTC, phenolic acids, and flavonoids) and antioxidant activities (DPPH, FRAP, ABTS, and TAC).

Variables	TPC	TFC	TTC	DPPH	ABTS	FRAP	TAC	Phenolic Acids
**TFC**	0.488 *							
**TTC**	0.932 **	0.457 *						
**DPPH**	0.718 **	0.396	0.720 **					
**ABTS**	0.591 **	0.270	0.622 **	0.904 **				
**FRAP**	0.722 **	0.314	0.603 **	0.868 **	0.835 **			
**TAC**	0.780 **	0.397	0.668 **	0.850 **	0.779 **	0.967 **		
**Phenolic acids**	0.496 *	0.343	0.515 *	0.761 **	0.628 *	0.614 **	0.640 **	
**Flavonoids**	0.349	0.232	0.355	0.633 *	0.535 *	0.473 *	0.452 *	0.911 **

* Significant correlation with *p* ≤ 0.05; ** Significant correlation with *p* ≤ 0.01. Phenolic acids and flavonoids are quantified through HPLC-PDA.

## Supplementary Materials

The following are available online at https://www.mdpi.com/2304-8158/9/9/1206/s1, Table S1: Characterization of phenolic compounds in different fruit peel samples by LC-ESI-QTOF-MS/MS, Figure S1: Characterization of phenolic compounds in different fruit peels in the negative mode of ionization by LC-ESI-QTOF-MS/MS, Figure S2: Characterization of phenolic compounds in different fruit peels in the positive mode of ionization by LC-ESI-QTOF-MS/MS.
