# Supplementary material for: Screening and Characterization of Phenolic Compounds and Their Antioxidant Capacity in Different Fruit Peels
Source: Foods. 2020 Sep 1;9(9):1206. doi: 10.3390/foods9091206 (PMC7556026; doi:10.3390/foods9091206)
Supplement: Supplementary file 1 [file foods-09-01206-s001.pdf]

foods

MDPI

Supplemental material

Screening and characterization of phenolic compounds and their antioxidant capacity in different fruit peels

Hafiz A. R. Suleria[1,2*], Colin J. Barrow[2] and Frank R. Dunshea[1,3]

[1] School of Agriculture and Food, Faculty of Veterinary and Agricultural Sciences, The University of Melbourne, Parkville 3010, VIC, Australia; hafiz.suleria@unimelb.edu.au (H.A.R.S.); fdunshea@unimelb.edu.au (F.R.D.)

[2] Centre for Chemistry and Biotechnology, School of Life and Environmental Sciences, Deakin University, Waurn Ponds, VIC 3217, Australia; colin.barrow@deakin.edu.au (C.J.B)

[3] Faculty of Biological Sciences, The University of Leeds, Leeds LS2 9JT, United Kingdom.

* Correspondence: hafiz.suleria@unimelb.edu.au; Tel: +61 470 439 670

Received: date; Accepted: date; Published: date

Abstract: Fruit peels have a diverse range of phytochemicals including carotenoids, vitamins, dietary fibres and phenolic compounds, some with remarkable antioxidant properties. Nevertheless, the comprehensive screening and characterization of the complex array of phenolic compounds in different fruit peels is limited. This study aimed to determine the polyphenol content and their antioxidant potential in twenty different fruit peel samples in ethanolic extraction, including their comprehensive characterization and quantification by the LC-MS/MS and HPLC. The obtained results showed that mango peel exhibited the highest phenolic content for TPC (27.51 ± 0.63 mg GAE/g), TFC (1.75 ± 0.08 mg QE/g) while the TTC (9.01 ± 0.20 mg CE/g) was slightly higher in avocado peel than mango peel (8.99 ± 0.13 mg CE/g). In terms of antioxidant potential, grapefruit peel had the highest radical scavenging capacities for the DPPH (9.17 ± 0.19 mg AAE/g), ABTS (10.79 ± 0.56 mg AAE/g), ferric reducing capacity in FRAP (9.22 ± 0.25 mg AA/g) and total antioxidant capacity, TAC (8.77 ± 0.34 mg AAE/g) compared to other fruit peel samples. Application of LC-ESI-QTOF-MS/MS tentatively identified and characterized a total of 176 phenolics including phenolic acids (49), flavonoids (86), lignans (11), stilbene (5) and other polyphenols (25) in all twenty peel samples. From HPLC-PDA quantification, mango peel sample showed significantly higher phenolic content, particularly for phenolic acids (gallic acid, 14.5 ± 0.4 mg/g) and flavonoids (quercetin, 11.9 ± 0.4 mg/g), as compared to other fruit peel samples. These results highlight the importance of fruit peels as a potential source of polyphenols. This study provides supportive information for utilization of different phenolic rich fruit peels as ingredients in the food, feed and nutraceutical.

Keywords: Fruit peels; polyphenols; phenolic acids; flavonoids; flavan-3-ols; hydrolysable and condensed tannins; antioxidant activities; LC-MS and HPLC.

Materials and Methods (Supplementary material)

2.1. Chemicals and Reagents

Most of the chemicals used for extraction and characterization were analytical grade and purchased from Sigma-Aldrich (Castle Hill, NSW, Australia). Folin-Ciocalteu's phenol reagent, gallic acid, L-ascorbic acid, vanillin, hexahydrate aluminium chloride, sodium phosphate, iron(III) chloride hexahydrate ($Fe[III]Cl_3.6H_2O$), hydrated sodium acetate, hydrochloric acid, ammonium molybdate, quercetin, catechin, 2,2′-diphenyl-1-picrylhy-drazyl (DPPH), 2,4,6tripyridyl-s-triazine (TPTZ), and 2,2′-azinobis-(3-ethylbenzothiazoline-6-sulfonic acid) (ABTS) were purchased from the Sigma-Aldrich (Castle Hill, NSW, Australia) for the estimation of polyphenols and antioxidant potential. Reference standards for the HPLC including gallic acid, protocatechuic acid, caftaric acid, *p*-hydroxybenzoic acid, chlorogenic acid, caffeic acid, syringic acid, coumaric acid, ferulic acid, sinapinic acid, catechin, epicatechin gallate, quercetin-3-galactoside, quercetin-3-glucuronide, quercetin-3-glucoside, quercetin, diosmin, kaempferol and kaempferol-3-glucoside were produced by Sigma-Aldrich (Castle Hill, NSW, Australia) for quantification proposes. Sodium carbonate anhydrous were purchased from Chem-Supply Pty Ltd. (Adelaide, SA, Australia) and 98% sulfuric acid were bought from RCI Labscan (Rongmuang, Thailand). HPLC and LC-MS grade reagents include methanol, ethanol, acetonitrile, formic acid and glacial acetic acid were purchased from Thermo Fisher Scientific Inc (Scoresby, VIC, AU). To perform various *in vitro* bioactivities and antioxidant assays, 96 well-plates were purchased from Thermo Fisher Scientific (VIC, Australia). Additionally, HPLC vials (1 mL) were purchased from Agilent technologies (VIC, Australia).

2.2. Sample Preparation

Twenty different Australian grown fresh and mature fruits varieties (2-3 kg) including apple (Royal gala), apricot (Mystery), avocado (Hass), banana (Cavendish), custard apple (African Pride), dragon fruit (Red-fleshed), grapefruit (Thompson), kiwifruit (Hayward), mango (Kensington Pride), lime (Tahitian), melon (Rock melons), nectarine (Fantasia), orange (Navels), papaya (Sunrise Solo), passionfruit (Misty Gem), peach (Florda gold), pear (Packham's Triumph), pineapple (Aussie Rough), plum (Angeleno), and pomegranate (Griffith) were purchased from a local produce market in Melbourne, Australia. The fruits were manually cleaned, peels were removed and cut into desirable slices (0.5 x 1 cm) and frozen at − 20 °C for overnight followed by lyophilization at − 45 °C/50 MPa using the Dynavac engineering FD3 Freeze Drier (Belmont, W.A., Australia) and Edwards RV12 oil sealed rotary vane pump (Bolton, England). The freeze-dried fruit peels were grounded into a refined powder by electric grinder (Sunbeam Multi Grinder - EM0405, Melbourne, VIC, AU), packed into silver flat Ziplock aluminum foil - vacuum sealing bags (Best supply, NSW, AU) and stored at − 20 °C.

2.3. Extraction of Phenolic Compounds

To extract the phenolic compounds, 2.0 ± 0.5 g of each fruit peel powder was mixed with 20 mL 70% ethanol. The samples were homogenized at 10, 000 rpm for 30 s using the IKA Ultra-Turrax T25 homogenizer (Rawang, Selangor, Malaysia) and subjected to shaking incubator (ZWYR-240, Labwit, Ashwood, VIC, Australia) at 120 rpm for 12 h (4 °C). After incubation, the extracts were centrifuged with Hettich Refrigerated Centrifuge (ROTINA380R, Tuttlingen, Baden-Württemberg, Germany) at 5, 000 rpm for 15 min. The supernatants were collected and stored at − 20 °C for 2 weeks for antioxidant analysis. For HPLC and LC-MS analysis, the extracts were filtrated through a 0.45 μm syringe filter (Thermo Fisher Scientific Inc., Waltham, MA, USA).

2.4. Estimation of Polyphenols and Antioxidant Potential

For polyphenol estimation in selected fruit peel samples, TPC, TFC, and TTC assays were performed while for measuring their antioxidant potential, four different types of antioxidant assays including DPPH, ABTS, FRAP and TAC were performed by adopting our previously published

methods of Tang, *et al.* [18]. The data was determined using a Multiskan® Go microplate photometer (Thermo Fisher Scientific, Waltham, MA, USA).

2.4.1. Determination of Total Phenolic Content (TPC)

For the TPC, 25 μL extracts of each peel extract, 200 μL of water and 25 μL of Folin–Ciocalteu reagent solution (1:3 v/v), diluted with water was added to 96 well plate (Corning Inc., Midland, NC, USA) followed by incubation at 25 °C for 5 minutes. After that, 25 μL 10% (w:w) sodium carbonate was added and incubated for 1 h at 25 °C followed by the measurement of absorbance at 765 nm by a spectrophotometer plate reader (Thermo Fisher Scientific, Waltham, MA, USA). The quantification of total phenolic content was based on a standard curve generated from gallic acid with the concentrations from 0 – 200 μg/mL and results were expressed as mass (mg) of gallic acid equivalents (GAE) per weight of sample.

2.4.2. Determination of Total Flavonoids Content (TFC)

For the TFC, 80 μL of each peel extract, 80 μL of 2% (w/v) aluminum chloride solution and 120 μL of 50 g/L sodium acetate solution were added in a 96-well plate followed by incubation at 25 °C for 2.5 h and absorbance was measured at 440 nm. For quantification, a standard curve was made with quercetin (0 – 50 μg/mL) and results were expressed as mass (mg) of quercetin equivalents (QE) per weight of sample.

2.4.3. Determination of Total Tannins Content (TTC)

For the TTC, 25 μL of extract, 150 μL 4% (w/v) vanillin solution and 25 μL of 32% (v/v) sulphuric acid were incubated at 25 °C for 15 min, absorbance was measured at 500 nm. For quantification, a standard curve was generated from catechin using the concentrations of 0 - 1000 μg/mL and results were expressed as mass (mg) of catechin equivalents (CE) per weight of sample.

2.4.4. Determination of 2,2′-Diphenyl-2-picryl-hydrazyl (DPPH) Antioxidant Assay

For the DDH assays, 40 μL of each fruit peel extract and 260 μL of 0.1 M DPPH radical methanol solution was added into 96-well plate and incubated at 25 °C for 30 min. The absorbance was measured at 517 nm using a microplate reader. A standard curve was generated using 0 - 50 μg/mL ascorbic acid aqueous solution. The results were expressed as mass (mg) of ascorbic acid equivalents (AAE) per weight of sample.

2.4.5. Determination of Ferric Reducing Antioxidant Power (FRAP) Assay

To prepare the FRAP reagent, 300 mM sodium acetate buffer (pH 3.6), 10 mM TPTZ solution, and 20 mM ferric chloride in a ratio of 10:1:1 (v/v/v) was prepared freshly. A 20 μL of peel extracts and 280 μL of freshly prepared FRAP reagent were mixed in a 96 well plate followed by incubation at 37 °C for 10 min, absorbance was measured at 593 nm. A standard curve was achieved using concentrations of 0 - 50 μg/mL ascorbic acid and results were expressed as mass (mg) of AAE per weight of sample.

2.4.6. Determination of 2,2′-azinobis-(3-ethylbenzothiazoline-6-sulfonic acid) (ABTS) Assay

The $ABTS^+$ dye was prepared with 5 mL of 7 mM of ABTS solution mixed with 88 μL of 140 mM potassium persulfate solution, incubated in the dark at room temperature for 16 h to generate an $ABTS^+$ free radical solution. Further, $ABTS^+$ stock solution was prepared by diluted with ethanol to gain absorbance of 0.70 at 734 nm. For the ABTS assay, 10 μL fruit peel extract and 290 μL of freshly prepared $ABTS^+$ solution were added in 96 well plate and incubated at 25 °C for 6 min. Subsequently, the absorbance was measured at 734 nm. A standard curve was achieved using concentrations of 0 - 150 μg/mL ascorbic acid and the results were expressed as mass (mg) of AAE per weight of sample.

2.4.7. Determination of Total Antioxidant Capacity (TAC)

For the TAC, 40 μL of each fruit peel extract was added to 260 μL of phosphomolybdate reagent (0.6 M H_2SO_4, 0.028 M sodium phosphate and 0.004 M ammonium molybdate). The mixture was incubated at 95 °C for 10 min, cooled at room temperature and absorbance was measured at 695 nm. A standard curve was generated using concentrations of 0 - 200 μg/mL ascorbic acid and the results were expressed as mass (mg) of AAE per weight of sample.

2.5. Characterization of Phenolic compounds using LC-ESI-QTOF-MS/MS Analysis

The phenolic compound characterization was performed on an Agilent 1200 HPLC with an Agilent 6520 Accurate- Mass Q-TOF LC/MS (Agilent Technologies, Santa Clara, CA, USA). The separation was conducted using a Synergi Hydro-RP 80 Å, reverse phase column (250 mm x 4.6 mm, 4 μm particle size) with protected C18 ODS (4.0 × 2.0 mm) guard column (Phenomenex, Lane Cove, NSW, Australia) by adopting our previously published method of Zhong, *et al.* [19]. In brief, the mobile phase consisted of water/acetic acid (98:2, v/v; eluent A) and acetonitrile/acetic acid/ water (50:0.5:49.5, v/v/v; eluent B). The gradient profile was described as follows: 10–25% B (0–25 min), 25–35% B (25–35 min), 35–40% B (35–45 min), 40–55% B (45–75 min), 55–80% B (75–79 min), 80–90% B (79–82 min), 90–100% B (82–84 min), 100–10% B (84–87 min), isocratic 10% B (87–90 min). A 6 μL of each peel extract was injected and the flow rate was set at 0.8 mL/min. Peaks were identified in both positive and negative ion modes with the capillary and nozzle voltage set to 3.5 kV and 500 V, respectively. Additionally, following conditions were maintained; i) nitrogen gas temperature at 300 °C, ii) sheath gas flow rate of 11 L/min at 250 °C, ii) nitrogen gas nebulisation at 45 psi. A complete mass scan ranging from *m/z* 50 to 1300 was used, MS/MS analyses were carried out in automatic mode with collision energy (10, 15 and 30 eV) for fragmentation. Peak identification was performed in both positive and negative modes while the instrument control, data acquisition and processing were performed using LC-ESI-QTOF-MS/MS MassHunter workstation software (Qualitative Analysis, version B.03.01, Agilent Technologies, Santa Clara, CA, USA).

2.6. Quantification of Phenolic compounds using HPLC-PDA

The quantitative measurement of targeted phenolic compounds present in different fruit peels samples was performed with an Agilent 1200 HPLC equipped with a photodiode array (PDA) detector by adopting our previously published protocol of Ma, *et al.* [20]. In brief, the same column and conditions were maintained as described above in LC-ESI-QTOF-MS/MS, except for a sample injection volume of 20 μL. The twenty most abundant phenolic compounds present in the different fruit peels including 10 phenolic acids and 10 flavonoids, were selected for quantification purposes. The phenolic compounds were determined at three different wavelengths, including 280 nm, 320 nm, and 370 nm. The quantification of targeted polyphenols was based on the calibration standard curve and the result was expressed as mg/g of sample. Data collection and processing was performed using Agilent MassHunter workstation software (Agilent Technologies, Santa Clara, CA, USA).

2.7. Statistical Analysis

All analyses were performed in triplicates and the results are presented as mean ± standard deviation (n = 3). The mean differences between different samples were analyzed by one-way analysis of variance (ANOVA) and Tukey's honestly significant differences (HSD) multiple rank test at $p \leq 0.05$. ANOVA was carried out by Minitab for Windows version 19.0 (Minitab, LLC, State College, PA, USA) and GraphPad Prism 7.05 Software for Windows (GraphPad 7.05 Software, San Diego, CA, USA, www.graphpad.com). For correlations between polyphenol content and antioxidant activities by Pearson's correlation coefficient at $p \leq 0.05$ and multivariate statistical analysis including principal component analysis (PCA), XLSTAT – 2019.1.3 were used by Addinsoft Inc. New York, N.Y USA.

Table S1. Characterization of phenolic compounds in different fruit peel samples by LC-ESI-QTOF-MS/MS.

No.	Proposed compounds	Molecular Formula	RT (min)	Ionization (ESI^+/ESI^-)	Molecular Weight	Theoretical (*m/z*)	Observed (*m/z*)	Error (ppm)	MS^2 Product ions	Fruit Peels
**Phenolic acid**										
**Hydroxybenzoic acids**										
1	Vanillic acid 4-sulfate	$C_8H_8O_7S$	5.068	$[M-H]^-$	247.9991	246.9918	246.9911	-2.8	167	*MNG, PER, KWF
2	Gallic acid 4-*O*-glucoside	$C_{13}H_{16}O_{10}$	6.866	$[M-H]^-$	332.0743	331.0670	331.0674	1.2	169, 125	*APL, APR, GRF, MNG, ORN, PSN, PER, PIN, PLM, POM
3	Gallic acid	$C_7H_6O_5$	6.873	$**[M-H]^-$	170.0215	169.0142	169.0146	2.4	125	*MNG, ORN, PER, POM, KWF, LMN
4	Ellagic acid arabinoside	$C_{19}H_{14}O_{12}$	7.020	$[M-H]^-$	434.0485	433.0412	433.0422	2.3	300	ORN
5	Protocatechuic acid 4-*O*-glucoside	$C_{13}H_{16}O_9$	7.379	$**[M-H]^-$	316.0794	315.0721	315.0718	-1.0	153	*APL, APR, BNA, GRF, KWF, MNG, ORN, PSN, PEC, PER, PIN, PLM, POM, AVO, PAP
6	2-Hydroxybenzoic acid	$C_7H_6O_3$	7.628	$**[M-H]^-$	138.0317	137.0244	137.0244	0.1	93	*APL, APR, BNA, GRF, KWF, MNG, NEC, PEC, PSN, PER, PIN, AVO, PAP
7	4-Hydroxybenzoic acid 4-*O*-glucoside	$C_{13}H_{16}O_8$	11.171	$[M-H]^-$	300.0845	299.0772	299.0762	-3.3	255, 137	*GRF, MNG, MEL, PER
8	2,3-Dihydroxybenzoic acid	$C_7H_6O_4$	12.714	$[M-H]^-$	154.0266	153.0193	153.0193	0.1	109	*APL, GRF, KWF, NEC, PEC, ORN, PSN, PIN, PLM
9	3-*O*-Methylgallic acid	$C_8H_8O_5$	13.079	$**[M+H]^+$	184.0372	185.0445	185.0452	3.8	170, 142	*KWF, MNG, AVO, DGF, GRF, PEC
10	3,4-*O*-Dimethylgallic acid	$C_9H_{10}O_5$	16.475	$**[M+H]^+$	198.0528	199.0601	199.0605	2.0	153, 139, 125, 111	*DGF, KWF, MNG, ORN, PAP, PEC, AVO, CTA
11	Gallic acid 3-*O*-gallate	$C_{14}H_{10}O_9$	21.104	$[M-H]^-$	322.0325	321.0252	321.0240	-3.7	169	*MNG, PER
12	Paeoniflorin	$C_{23}H_{28}O_{11}$	58.033	$**[M-H]^-$	480.1632	479.1559	479.1577	3.8	449, 357, 327	*LMN, AVO, DGF
**Hydroxycinnamic acids**										
13	1,5-Dicaffeoylquinic acid	$C_{25}H_{24}O_{12}$	4.134	$**[M-H]^-$	516.1268	515.1195	515.1198	0.6	353, 335, 191, 179	*NEC, ORN, PSN, AVO, CTA
14	Isoferulic acid 3-sulfate	$C_{10}H_{10}O_7S$	5.341	$[M-H]^-$	274.0147	273.0074	273.0067	-2.6	193, 178	PLM
15	Caffeoyl glucose	$C_{15}H_{18}O_9$	7.012	$[M-H]^-$	342.0951	341.0878	341.0861	-5.0	179, 161	*BNA, DGF, GRF, KWF, NEC, ORN, PSN, PLM, POM
16	*p*-Coumaroyl tartaric acid	$C_{13}H_{12}O_8$	8.632	$**[M-H]^-$	296.0532	295.0459	295.0468	3.1	115	*AVO, DGF, PIN, GRF, LMN, ORN, PER
17	Cinnamic acid	$C_9H_8O_2$	9.351	$**[M-H]^-$	148.0524	147.0451	147.0448	-2.0	103	*APL, APR, BNA, CTA, LMN, PEC, PER, PIN, PLM, POM, AVO, DGF, MEL
18	Feruloyl tartaric acid	$C_{14}H_{14}O_9$	10.419	$[M-H]^-$	326.0638	325.0565	325.0566	0.3	193, 149	*MNG, PER, POM
19	Caffeoyl tartaric acid	$C_{13}H_{12}O_9$	13.756	$**[M-H]^-$	312.0481	311.0408	311.0418	3.2	161	*POM, MNG, ORN, PSN
20	3-Sinapoylquinic acid	$C_{18}H_{22}O_{10}$	14.154	$**[M-H]^-$	398.1213	397.1140	397.1144	1.0	233, 179	*CTA, NEC, ORN, AVO, DGF, PAP
21	3-*p*-Coumaroylquinic acid	$C_{16}H_{18}O_8$	18.131	$**[M-H]^-$	338.1002	337.0929	337.0924	-1.5	265, 173, 162	*APL, APR, CTA, KWF, NEC, PEC, PSN, PLM, AVO, DRF, MEL

22	Ferulic acid 4-*O*-glucoside	$C_{16}H_{20}O_9$	18.495	**[M-H]$^-$	356.1107	355.1034	355.1024	-2.8	193, 178, 149, 134	*APR, KWF, MNG, NEC, PIN, PLM, POM, AVO, CTA, PAP
23	Ferulic acid	$C_{10}H_{10}O_4$	18.512	**[M-H]$^-$	194.0579	193.0506	193.0500	-3.1	178, 149, 134	*APR, KWF, NEC, PSN, PLM, AVO, DGF, PAP
24	Hydroxycaffeic acid	$C_9H_8O_5$	19.279	[M-H]$^-$	196.0372	195.0299	195.0294	-2.6	151	*ORN, PEC, PLM
25	*m*-coumaric acid	$C_9H_8O_3$	19.319	**[M-H]$^-$	164.0473	163.0400	163.0406	3.7	119	*APL, APR, BNA, CTA, GRF, KWF, NEC, PEC, PSN, PIN, PLM, POM, AVO, DGF, PAP
26	Caffeic acid 3-*O*-glucuronide	$C_{15}H_{16}O_{10}$	19.588	**[M-H]$^-$	356.0743	355.0670	355.0686	4.5	179	*CTA. GRF, KWF, ORN, PIN, DGF
27	Ferulic acid 4-*O*-glucuronide	$C_{16}H_{18}O_{10}$	19.704	**[M-H]$^-$	370.0900	369.0827	369.0834	1.9	193	*APR, AVO, CTA, GRF, KWF, ORN, PSN, PLM, LMN, MNG, POM
28	Caffeic acid 4-sulfate	$C_9H_8O_7S$	20.240	[M-H]$^-$	259.9991	258.9918	258.9916	-0.8	179, 135	ORN
29	3-Caffeoylquinic acid	$C_{16}H_{18}O_9$	20.815	**[M-H]$^-$	354.0951	353.0878	353.0877	-0.3	253, 190, 144	*APL, APR, CTA, DGF, KWF, PEC, ORN, PSN, PLM, AVO, LMN, PAP
30	*p*-Coumaric acid 4-*O*-glucoside	$C_{15}H_{18}O_8$	20.881	[M-H]$^-$	326.1002	325.0929	325.0925	-1.2	163	*APL, AVO, GRF, KWF, MNG, PEC, PLM, POM
31	*p*-Coumaroyl tyrosine	$C_{18}H_{17}NO_5$	25.148	[M-H]$^-$	327.1107	326.1034	326.1035	0.3	282	DGF
32	1-Sinapoyl-2,2'-diferuloylgentiobiose	$C_{43}H_{48}O_{21}$	26.763	[M-H]$^-$	900.2688	899.2615	899.2579	-4.0	613, 201	KWF
33	Sinapic acid	$C_{11}H_{12}O_5$	30.185	**[M-H]$^-$	224.0685	223.0612	223.0603	-4.0	205, 163	*AVO, CTA, APL, KWF, PAP. LMN, PIN
34	Caffeic acid	$C_9H_8O_4$	31.284	**[M-H]$^-$	180.0423	179.0350	179.0349	-0.6	143, 133	*CTA, GRF, NEC, ORN, PSN, PLM, PAP, PER, PIN
35	Verbascoside	$C_{29}H_{36}O_{15}$	31.531	[M-H]$^-$	624.2054	623.1981	623.1984	0.4	477, 461, 315, 135	*CTA, DGF, LMN
36	5-5'-Dehydrodiferulic acid	$C_{20}H_{18}O_8$	32.124	[M+H]$^+$	386.1002	387.1075	387.1064	-2.8	369	*DGF, KWF
37	Rosmarinic acid	$C_{18}H_{16}O_8$	32.802	[M-H]$^-$	360.0845	359.0772	359.0787	4.2	179	*AVO, CTA, DGF, KWF, PER
38	3-Feruloylquinic acid	$C_{17}H_{20}O_9$	33.605	**[M-H]$^-$	368.1107	367.1034	367.1019	-4.1	298, 288, 192, 191	*APL, APR, CTA, MNG, NEC, PEC, ORN, PSN, PER, PLM, AVO, DGF, MEL
39	1,2,2'-Triferuloylgentiobiose	$C_{42}H_{46}O_{20}$	34.101	[M-H]$^-$	870.2582	869.2509	869.2498	-1.3	693, 517	PAP
40	Chicoric acid	$C_{22}H_{18}O_{12}$	35.138	[M−H]$^-$	474.0798	473.0725	473.0754	3.1	293, 311	*DGF, KWF
41	1-Sinapoyl-2-feruloylgentiobiose	$C_{33}H_{40}O_{18}$	36.370	[M-H]$^-$	724.2215	723.2142	723.2124	-2.5	529, 499	APR
42	*p*-Coumaroyl malic acid	$C_{13}H_{12}O_7$	41.506	[M-H]$^-$	280.0583	279.0510	279.0524	5.0	163, 119	PAP
43	Cinnamoyl glucose	$C_{15}H_{18}O_7$	60.985	**[M-H]$^-$	310.1053	309.0980	309.0965	-4.9	147, 131, 103	*PER, DGF
**Hydroxyphenylacetic acids**										
44	3,4-Dihydroxyphenylacetic acid	$C_8H_8O_4$	20.749	**[M-H]$^-$	168.0423	167.035	167.0343	-4.2	149, 123	*APL, APR, CTA, GRF, MNG, MEL, NEC, PEC, ORN, PSN, PER, PIN, PLM, POM, AVO, DGF
45	2-Hydroxy-2-phenylacetic acid	$C_8H_8O_3$	36.121	**[M-H]$^-$	152.0473	151.0400	151.0407	4.6	136, 92	*CTA, KWF, MNG, ORN, PER, DGF
**Hydroxyphenylpropanoic acids**										

46	Dihydroferulic acid 4-sulfate	$C_{10}H_{12}O_7S$	4.076	$[M-H]^-$	276.0304	275.0231	275.0229	-0.7	195, 151, 177	AVO
47	Dihydroferulic acid 4-*O*-glucuronide	$C_{16}H_{20}O_{10}$	6.866	$[M-H]^-$	372.1056	371.0983	371.0986	0.8	195	*APL, APR, CTA, KWF, NEC, ORN, PSN, PLM
48	3-Hydroxy-3-(3-hydroxyphenyl) propionic acid	$C_9H_{10}O_4$	10.956	$[M-H]^-$	182.0579	181.0506	181.0500	-3.3	163, 135, 119	*GRF, MNG, ORN, PEC, PER
49	Dihydrocaffeic acid 3-*O*-glucuronide	$C_{15}H_{18}O_{10}$	22.536	$[M-H]^-$	358.090	357.0827	357.0811	-4.5	181	*GRF, PEC, PER, PIN, POM
**Flavonoids**										
**Flavanols**										
50	Prodelphinidin dimer B3	$C_{30}H_{26}O_{14}$	16.428	$**[M+H]^+$	610.1323	611.1396	611.1367	-4.7	469, 311, 291	*CTA, KWF, PEC, POM, AVO, DGF
51	(+)-Catechin 3-*O*-gallate	$C_{22}H_{18}O_{10}$	22.306	$**[M-H]^-$	442.090	441.0827	441.0805	-5.0	289, 169, 125	*KWF, PER, AVO
52	(-)-Epigallocatechin	$C_{15}H_{14}O_7$	24.121	$**[M-H]^-$	306.0740	305.0667	305.0675	2.6	261, 219	AVO
53	3'-*O*-Methylcatechin	$C_{16}H_{16}O_6$	24.124	$**[M-H]^-$	304.0947	303.0874	303.0878	1.3	271, 163	*PER, AVO, LMN
54	(+)-Catechin	$C_{15}H_{14}O_6$	26.597	$**[M-H]^-$	290.0790	289.0717	289.0706	-3.8	245, 205, 179	*APL, APR, CTA, GRF, KWF, MNG, PSN, PEC, PER, PLM, POM, AVO, DGF, PAP
55	4''-*O*-Methylepigallocatechin 3-*O*-gallate	$C_{23}H_{20}O_{11}$	27.887	$**[M-H]^-$	472.1006	471.0933	471.0923	-2.1	169, 319	*GRF, POM, AVO
56	Procyanidin trimer C1	$C_{45}H_{38}O_{18}$	28.966	$**[M-H]^-$	866.2058	865.1985	865.1961	-2.8	739, 713, 695	*APL, CTA, KWF, MNG, PAP, PEC, PLM, POM, AVO, DGF
57	(+)-Gallocatechin 3-*O*-gallate	$C_{22}H_{18}O_{11}$	29.655	$[M-H]^-$	458.0849	457.0776	457.0777	0.2	305, 169	*AVO, PAP
58	4'-*O*-Methyl-(-)-epigallocatechin 7-*O*-glucuronide	$C_{22}H_{24}O_{13}$	31.732	$[M-H]^-$	496.1217	495.1144	495.1123	-4.2	451, 313	*APL, NEC, PEC, AVO, KWF, PER, PLM
59	Cinnamtannin A2	$C_{60}H_{50}O_{24}$	35.276	$**[M-H]^-$	1154.269	1153.262	1153.2600	-1.8	739	*CTA, KWF, PLM, AVO, DGF
60	Procyanidin dimer B1	$C_{30}H_{26}O_{12}$	37.978	$**[M-H]^-$	578.1424	577.1351	577.1348	-0.5	451	*APL, AVO, CTA, GRF, KWF, NEC, PEC, ORN, PLM, POM, DGF, PAP
**Flavones**										
61	Apigenin 7-*O*-(6''-malonyl-apiosyl-glucoside)	$C_{29}H_{30}O_{17}$	4.416	$[M-H]^-$	650.1483	649.1410	649.1429	2.9	605	PEC
62	Gardenin B	$C_{19}H_{18}O_7$	10.234	$**[M+H]^+$	358.1053	359.1126	359.1118	-2.2	344, 329, 311	*CTA, AVO, BNA
63	Cirsilineol	$C_{18}H_{16}O_7$	10.827	$**[M+H]^+$	344.0896	345.0969	345.0970	0.3	330, 312, 297, 284	*DGF, BNA, KWF, LMN
64	7,4'-Dihydroxyflavone	$C_{15}H_{10}O_4$	18.251	$[M+H]^+$	254.0579	255.0652	255.0643	-3.5	227, 199, 171	*AVO, PER, PIN
65	Apigenin 7-*O*-glucuronide	$C_{21}H_{18}O_{11}$	20.967	$**[M+H]^+$	446.0849	447.0922	447.0910	-2.7	271, 253	*CTA, DGF, PAP, KWF
66	Rhoifolin	$C_{27}H_{30}O_{14}$	27.229	$**[M-H]^-$	578.1636	577.1563	577.1538	-4.3	413, 269	PSN, LMN
67	Apigenin 7-*O*-apiosylglucoside	$C_{26}H_{28}O_{14}$	35.572	$**[M+H]^+$	564.1479	565.1552	565.1529	-4.1	296	*LMN, KWF, MNG, PAP
68	Apigenin 6,8-di-C-glucoside	$C_{27}H_{30}O_{15}$	43.578	$**[M-H]^-$	594.1585	593.1512	593.1527	2.5	503, 473	*APL, APR, GRF, KWF, ORN, PAP, PSN, PEC, PLM, LMN, MEL, PAP
69	Diosmin	$C_{28}H_{32}O_{15}$	46.538	$[M+H]^+$	608.1741	609.1814	609.1788	-4.3	301, 286	LMN

70	6-Hydroxyluteolin 7-rhamnoside	$C_{21}H_{20}O_{11}$	46.758	**[M-H]-	448.1006	447.0933	447.0928	-1.1	301	*APL, APR, BNA, DGF, KWF, ORN, PSN, PEC, PER, PLM, POM, AVO, LMN, MEL, PAP
71	Chrysoeriol 7-*O*-glucoside	$C_{22}H_{22}O_{11}$	54.226	**[M+H]+	462.1162	463.1235	463.1255	4.3	445, 427, 409, 381	*AVO, APL, KWF, POM, LMN
72	Apigenin 6-C-glucoside	$C_{21}H_{20}O_{10}$	55.754	**[M-H]-	432.1056	431.0983	431.0983	0.1	413, 341, 311	*APL, DGF, LMN, MNG, PLM
	**Flavanones**									
73	Hesperetin 3'-sulfate	$C_{16}H_{14}O_9S$	6.681	**[M-H]-	382.0359	381.0286	381.0293	1.8	301, 286, 257, 242	*GRF, CTA
74	Hesperetin 3',7-*O*-diglucuronide	$C_{28}H_{30}O_{18}$	21.163	**[M-H]-	654.1432	653.1359	653.1361	0.3	477, 301, 286, 242	*KWF, PIN, PAP
75	6-Prenylnaringenin	$C_{20}H_{20}O_5$	35.742	[M+H]+	340.1311	341.1384	341.1375	-2.6	323, 137	AVO
76	Narirutin	$C_{27}H_{32}O_{14}$	38.326	**[M-H]-	580.1792	579.1719	579.1710	-1.6	271	*APL, NEC, DGF, LMN
77	Neoeriocitrin	$C_{27}H_{32}O_{15}$	39.899	**[M-H]-	596.1741	595.1668	595.1684	2.7	431, 287	*CTA, LMN, NEC, AVO, DGF
78	Hesperidin	$C_{28}H_{34}O_{15}$	42.745	[M+H]+	610.1898	611.1971	611.1956	-2.5	593, 465, 449, 303	LMN
79	Hesperetin 3'-*O*-glucuronide	$C_{22}H_{22}O_{12}$	47.521	**[M-H]-	478.1111	477.1038	477.1033	-1.0	301, 175, 113, 85	*APL, BNA, KWF, MNG, ORN, NEC, PEC, POM, AVO, LMN
80	Naringin 4'-*O*-glucoside	$C_{33}H_{42}O_{19}$	53.036	[M-H]-	742.2320	741.2247	741.2249	0.3	433, 271	CTA
	**Flavonols**									
81	Myricetin 3-*O*-rutinoside	$C_{27}H_{30}O_{17}$	8.156	**[M-H]-	626.1483	625.1410	625.1423	2.1	301	*LMN, MNG, NEC, PEC, PSN, POM, AVO
82	Quercetin 3'-*O*-glucuronide	$C_{21}H_{18}O_{13}$	12.512	**[M-H]-	478.0747	477.0674	477.0670	-0.8	301	*LMN, ORN, POM, KWF
83	Myricetin 3-*O*-arabinoside	$C_{20}H_{18}O_{12}$	16.496	**[M-H]-	450.0798	449.0725	449.0716	-2.0	317	*ORN, LMN
84	3-Methoxysinensetin	$C_{21}H_{22}O_8$	16.528	**[M+H]+	402.1315	403.1388	403.1395	1.7	388, 373, 355, 327	*AVO, BNA, MNG, NEC, PLM, CTA
85	3-Methoxynobiletin	$C_{22}H_{24}O_9$	17.999	**[M+H]+	432.1420	433.1493	433.1488	-1.2	403, 385, 373, 345	*DGF, PAP, PER
86	Myricetin 3-*O*-galactoside	$C_{21}H_{20}O_{13}$	19.288	[M-H]-	480.0904	479.0831	479.0810	-4.4	317	*BNA, ORN, POM
87	Patuletin 3-*O*-glucosyl-(1->6)-[apiosyl(1->2)]-glucoside	$C_{33}H_{40}O_{22}$	26.768	[M-H]-	788.2011	787.1938	787.1960	2.8	625, 463, 301, 271	ORN
88	Isorhamnetin	$C_{16}H_{12}O_7$	27.076	**[M-H]-	316.0583	315.0510	315.0504	-1.9	300, 271	*PLM, AVO, LMN, PAP
89	Spinacetin 3-*O*-(2	$C_{43}H_{48}O_{24}$	33.242	[M-H]-	948.2536	947.2463	947.2456	-0.7	741, 609, 301	PSN
90	Isorhamnetin 3-*O*-glucuronide	$C_{22}H_{20}O_{13}$	34.082	[M-H]-	492.0904	491.0831	491.0875	3.9	315, 300, 272, 255	*AVO, KWF
91	Quercetin 3-*O*-glucosyl-xyloside	$C_{26}H_{28}O_{16}$	36.319	[M-H]-	596.1377	595.1304	595.1311	1.2	265, 138, 116	*GRF, KWF, LMN, NEC, ORN, PLM
92	Kaempferol 3,7-*O*-diglucoside	$C_{27}H_{30}O_{16}$	37.879	**[M-H]-	610.1534	609.1461	609.1451	-1.6	447, 285	*APL, APR, NEC, PEC, ORN, PSN, PIN, PLM, LMN, PAP

93	Quercetin 3-*O*-xylosyl-rutinoside	$C_{32}H_{38}O_{20}$	39.018	**[M+H]$^{+}$	742.1956	743.2029	743.2060	4.2	479, 317	*DGF, AVO, CTA, PAP
94	Kaempferol 3-*O*-glucosyl-rhamnosyl-galactoside	$C_{33}H_{40}O_{20}$	40.181	**[M-H]$^{-}$	756.2113	755.204	755.2004	-4.8	285	*APL, AVO, MEL, ORN, PSN, PEC, PIN, PLM, POM, LMN
95	Kaempferol 3-*O*-(2''-rhamnosyl-galactoside) 7-*O*-rhamnoside	$C_{33}H_{40}O_{19}$	41.953	**[M-H]$^{-}$	740.2164	739.2091	739.2088	-0.4	593, 447, 285	*APR, AVO, LMN, ORN, PAP, PIN, PLM, POM
96	Quercetin 3-*O*-xylosyl-glucuronide	$C_{26}H_{26}O_{17}$	43.207	**[M+H]$^{+}$	610.1170	611.1243	611.1255	2.0	479, 303, 285, 239	*KWF, GRF, AVO
97	Myricetin 3-*O*-rhamnoside	$C_{21}H_{20}O_{12}$	44.025	**[M-H]$^{-}$	464.0955	463.0882	463.0881	-0.2	317	*APL, BNA, NEC, PEC, ORN, PSN, PEC, PLM, POM, LMN, PAP
98	Quercetin 3-*O*-arabinoside	$C_{20}H_{18}O_{11}$	46.344	**[M-H]$^{-}$	434.0849	433.0776	433.0776	0.1	301	*APL, GRF, MNG, ORN, PEC, PLM, CTA, DGF, PAP
99	Quercetin 3-*O*-(6''-malonyl-glucoside)	$C_{24}H_{22}O_{15}$	48.691	[M+H]$^{+}$	550.0959	551.1032	551.1074	4.62	303	*CTA, APL, ORN
**Dihydrochalcones**										
100	3-Hydroxyphloretin 2'-*O*-xylosyl-glucoside	$C_{26}H_{32}O_{15}$	37.564	[M-H]$^{-}$	584.1741	583.1668	583.1665	-0.5	289	*APL, MNG, PER, PIN
101	3-Hydroxyphloretin 2'-*O*-glucoside	$C_{21}H_{24}O_{11}$	43.048	**[M-H]$^{-}$	452.1319	451.1246	451.1258	2.7	289, 273	*APL, AVO, CTA, DGF, GRF, KWF, MNG, PAP, PER
102	Phloridzin	$C_{21}H_{24}O_{10}$	51.613	**[M-H]$^{-}$	436.1369	435.1296	435.1284	-2.8	273	*APL, CTA, KWF, ORN, PEC, POM, AVO, DGF, PAP
**Dihydroflavonols**										
103	Dihydromyricetin 3-*O*-rhamnoside	$C_{21}H_{22}O_{12}$	21.710	**[M-H]$^{-}$	466.1111	465.1038	465.1021	-3.7	301	*APL, AVO, CTA, KWF, NEC, PEC, PSN, PLM, POM, DGF
104	Dihydroquercetin	$C_{15}H_{12}O_{7}$	31.135	**[M-H]$^{-}$	304.0583	303.0510	303.0504	-2.0	285, 275, 151	*CTA, KWF, MNG, PEC, PER, PAP
**Anthocyanins**										
105	Cyanidin 3-*O*-diglucoside-5-*O*-glucoside	$C_{33}H_{41}O_{21}$	21.567	**[M+H]$^{+}$	773.2140	774.2213	774.2216	0.4	610, 464	*PAP, LMN, DGF
106	Cyanidin 3-*O*-(6''-*p*-coumaroyl-glucoside)	$C_{30}H_{27}O_{13}$	22.205	**[M+H]$^{+}$	595.1452	596.1525	596.1553	4.7	287	*KWF, APL, MNG, PEC, PER, PLM, POM, DGF, CTA, AVO, PAP
107	Delphinidin 3-*O*-xyloside	$C_{20}H_{19}O_{11}$	25.983	**[M-H]$^{-}$	435.0927	434.0854	434.0860	1.4	303	*MEL, CTA, KWF
108	Petunidin 3-*O*-(6''-acetyl-glucoside)	$C_{24}H_{25}O_{13}$	27.386	[M+H]$^{+}$	521.1295	522.1368	522.1358	-1.9	317	MEL
109	Isopeonidin 3-*O*-arabinoside	$C_{21}H_{21}O_{10}$	29.965	[M+H]$^{+}$	433.1135	434.1208	434.1213	1.1	271, 253, 243	*MNG, DGF
110	Delphinidin 3-*O*-glucosyl-glucoside	$C_{27}H_{31}O_{17}$	36.884	**[M+H]$^{+}$	627.1561	628.1634	628.1636	0.3	465, 3030	AVO
111	Peonidin 3-*O*-sambubioside-5-*O*-glucoside	$C_{33}H_{41}O_{20}$	37.050	**[M+H]$^{+}$	757.2191	758.2264	758.2263	-0.1	595, 449, 287	*AVO, LMN, PAP
112	Cyanidin 3-*O*-(2-*O*-(6-*O*-(E)-caffeoyl-D glucoside)-D-glucoside)-5-*O*-D-glucoside	$C_{43}H_{49}O_{24}$	39.696	[M+H]$^{+}$	949.2614	950.2687	950.2690	0.3	787, 463, 301	*APL, MNG, ORN, PEC, PER, POM
113	Cyanidin 3,5-*O*-diglucoside	$C_{27}H_{31}O_{16}$	42.367	**[M+H]$^{+}$	611.1612	612.1685	612.1664	-3.4	449, 287	*AVO, CTA, KWF, LMN, PAP, PEC, DGF
114	Delphinidin 3-*O*-glucoside	$C_{21}H_{21}O_{12}$	45.066	**[M+H]$^{+}$	465.1033	466.1106	466.1114	1.7	303	*CTA, AVO, DGF, KWF, PAP, POM

115	4-*O*-Methyldelphinidin 3-*O*-D-glucoside	$C_{22}H_{23}O_{12}$	48.482	$[M+H]^+$	479.1190	480.1263	480.1257	-1.2	317, 303, 285, 271	*DGF, AVO
116	Pelargonidin 3-*O*-rutinoside	$C_{27}H_{31}O_{14}$	50.950	$[M+H]^+$	579.1714	580.1787	580.1814	4.6	271, 433	LMN
**Isoflavonoids**										
117	6''-*O*-Malonylglycitin	$C_{25}H_{24}O_{13}$	7.256	**$[M+H]^+$	532.1217	533.1290	533.1286	-0.8	285, 270, 253	*PAP, POM
118	Sativanone	$C_{17}H_{16}O_5$	9.333	$[M-H]^-$	300.0998	299.0925	299.0932	2.3	284, 269, 225	CTA
119	2',7-Dihydroxy-4',5'-dimethoxyisoflavone	$C_{17}H_{14}O_6$	10.651	**$[M+H]^+$	314.0790	315.0863	315.0868	1.5	300, 282	MNG
120	Dihydrobiochanin A	$C_{16}H_{14}O_5$	15.236	$[M+H]^+$	286.0841	287.0914	287.0911	-1.0	269, 203, 201, 175	*AVO, CTA, KWF
121	6''-*O*-Malonyldaidzin	$C_{24}H_{22}O_{12}$	16.246	**$[M+H]^+$	502.1111	503.1184	503.1200	3.2	255	*AVO, PSN
122	Glycitin	$C_{22}H_{22}O_{10}$	20.950	**$[M+H]^+$	446.1213	447.1286	447.1294	1.8	285	*CTA, PER
123	Equol	$C_{15}H_{14}O_3$	21.803	$[M+H]^+$	242.0943	243.1016	243.1019	1.2	255, 211, 197	LMN
124	Violanone	$C_{17}H_{16}O_6$	25.419	**$[M-H]^-$	316.0947	315.0874	315.0875	0.3	300, 285, 135	*CTA, ORN, PLM, AVO, DGF, LMN
125	2'-Hydroxyformononetin	$C_{16}H_{12}O_5$	28.896	$[M+H]^+$	284.0685	285.0758	285.0760	0.7	270, 229	LMN
126	6''-*O*-Acetyldaidzin	$C_{23}H_{22}O_{10}$	29.504	**$[M-H]^-$	458.1213	457.1140	457.1121	-4.2	221	*MNG, PLM, DGF, PAP
127	Dalbergin	$C_{16}H_{12}O_4$	30.324	$[M-H]^-$	268.0736	267.0663	267.0644	-4.1	252, 224, 180	*DGF, AVO
128	3',4',7-Trihydroxyisoflavanone	$C_{15}H_{12}O_5$	31.267	**$[M-H]^-$	272.0685	271.0612	271.0605	-2.6	177, 151, 119, 107,	*CTA, GRF, PSN, PER, DGF, KWF, LMN
129	Formononetin 7-*O*-glucuronide	$C_{22}H_{20}O_{10}$	42.450	**$[M-H]^-$	444.1056	443.0983	443.0973	-2.3	267, 252	*PAP, AVO, DGF, LMN
130	5,6,7,3',4'-Pentahydroxyisoflavone	$C_{15}H_{10}O_7$	42.893	**$[M+H]^+$	302.0427	303.0500	303.0487	-4.3	285, 257	*KWF, MNG, NEC, PEC, ORN, PAP, PLM, AVO, DGF, LMN, PAP, APL, BNA, CTA
131	6''-*O*-Acetylglycitin	$C_{24}H_{24}O_{11}$	43.656	**$[M+H]^+$	488.1319	489.1392	489.1413	4.3	285, 270	*DGF, PAP, LMN
132	3'-Hydroxygenistein	$C_{15}H_{10}O_6$	51.410	**$[M+H]^+$	286.0477	287.0550	287.0557	2.4	269, 259	*AVO, CTA, LMN, PAP, GRF, PLM, POM
133	6''-*O*-Malonylgenistin	$C_{24}H_{22}O_{13}$	64.297	$[M+H]^+$	518.1060	519.1133	519.1157	4.6	271	AVO
134	2-Dehydro-*O*-desmethylangolensin	$C_{15}H_{12}O_4$	77.381	$[M-H]^-$	256.0736	255.0663	255.0656	-2.7	135, 119	MNG
135	3'-Hydroxydaidzein	$C_{15}H_{10}O_5$	82.152	$[M+H]^+$	270.0528	271.0601	271.0588	-4.8	253, 241, 225	*APR, CTA, PIN
**Other polyphenols**										
**Hydroxycoumarins**										
136	Esculin	$C_{15}H_{16}O_9$	13.406	$[M+H]^+$	340.0794	341.0867	341.0862	-1.4	179, 151	APR
137	Esculetin	$C_9H_6O_4$	27.821	$[M-H]^-$	178.0266	177.0193	177.0199	3.4	149, 133, 89	CTA
138	Coumarin	$C_9H_6O_2$	32.744	**$[M+H]^+$	146.0368	147.0441	147.0448	4.8	103, 91	*AVO, PLM
139	Scopoletin	$C_{10}H_8O_4$	36.851	**$[M-H]^-$	192.0423	191.0350	191.0345	-2.6	176	*APR, DGF, LMN
140	Urolithin A	$C_{13}H_8O_4$	75.771	$[M-H]^-$	228.0423	227.0350	227.0341	-3.9	198, 182	*PSN, GRF, PLM
**Hydroxybenzaldehydes**										

141	*p*-Anisaldehyde	$C_8H_8O_2$	13.53	**[M+H]+	136.0524	137.0597	137.0597	0.1	122, 109	*AVO, APR, DGF, KWF, ORN, PAP, PSN, PLM, CTA, NEC, PEC, PER
142	4-Hydroxybenzaldehyde	$C_7H_6O_2$	44.568	**[M-H]-	122.0368	121.0295	121.0301	5.0	77	*BNA, GRF, PSN, PEC, PER, PIN, PLM, POM, AVO, PAP
**Hydroxybenzoketones**										
143	2-Hydroxy-4-methoxyacetophenone 5-sulfate	$C_9H_{10}O_7S$	9.446	[M-H]-	262.0147	261.0074	261.0067	-2.7	181, 97	PER
144	2,3-Dihydroxy-1-guaiacylpropanone	$C_{10}H_{12}O_5$	33.57	**[M-H]-	212.0685	211.0612	211.0605	-3.3	167, 123, 105, 93	*CTA, PIN, APR, AVO, DGF, MNG, PAP, PSN
**Hydroxyphenylpropenes**										
145	2-Methoxy-5-prop-1-enylphenol	$C_{10}H_{12}O_2$	26.251	[M+H]+	164.0837	165.0910	165.0902	-4.8	149, 137, 133, 124	AVO
**Curcuminoids**										
146	Curcumin	$C_{21}H_{20}O_6$	22.918	[M-H]-	368.126	367.1187	367.1207	4.4	217	*KWF, DGF
147	Bisdemethoxycurcumin	$C_{19}H_{16}O_4$	77.677	[M+H]+	308.1049	309.1122	309.1137	4.9	291, 263	DGF
148	Demethoxycurcumin	$C_{20}H_{18}O_5$	81.976	[M-H]-	338.1154	337.1081	337.1080	-0.3	217	BNA
**Furanocoumarins**										
149	Isopimpinellin	$C_{13}H_{10}O_5$	28.172	[M+H]+	246.0528	247.0601	247.0613	4.9	232, 217, 205, 203	*AVO, BNA, CTA
**Phenolic terpenes**										
150	Rosmanol	$C_{20}H_{26}O_5$	22.230	[M+H]+	346.1780	347.1853	347.1844	-2.6	301, 241, 231	PAP
151	Carnosic acid	$C_{20}H_{28}O_4$	80.419	**[M-H]-	332.1988	331.1915	331.1905	-3.0	287, 269	*BNA, LMN, AVO
**Tyrosols**										
152	3,4-DHPEA-AC	$C_{10}H_{12}O_4$	11.802	**[M-H]-	196.0736	195.0663	195.0657	-3.1	135	*APR, AVO, KWF, MEL, PIN, DGF, LMN, MNG, PAP
153	Hydroxytyrosol 4-*O*-glucoside	$C_{14}H_{20}O_8$	12.805	**[M-H]-	316.1158	315.1085	315.1092	2.2	153, 123	*DGF, KWF, MNG, ORN, PER, POM, AVO
154	Oleoside 11-methylester	$C_{17}H_{24}O_{11}$	17.600	[M-H]-	404.1319	403.1246	403.1269	4.7	223, 165	*CTA, AVO, DGF, KWF
155	3,4-DHPEA-EDA	$C_{17}H_{20}O_6$	23.564	[M-H]-	320.126	319.1187	319.1189	0.6	275, 195	*AVO, DGF
156	Demethyloleuropein	$C_{24}H_{30}O_{13}$	51.646	**[M-H]-	526.1686	525.1613	525.1599	-2.7	495	*APL, CTA, AVO, MEL
**Other polyphenols**										
157	Lithospermic acid	$C_{27}H_{22}O_{12}$	5.051	[M-H]-	538.1111	537.1038	537.1048	1.9	493, 339, 295	*MNG, PER, KWF
158	Arbutin	$C_{12}H_{16}O_7$	5.129	**[M-H]-	272.0896	271.0823	271.0828	1.8	109	*PSN, AVO
159	Salvianolic acid B	$C_{36}H_{30}O_{16}$	28.598	[M-H]-	718.1534	717.1461	717.1436	-3.5	519, 339, 321, 295	BNA
160	Salvianolic acid C	$C_{26}H_{20}O_{10}$	32.51	[M-H]-	492.1056	491.0983	491.0993	2.0	311, 267, 249	*CTA, PAP
**Lignans**										
161	Enterolactone	$C_{18}H_{18}O_4$	4.254	[M+H]+	298.1205	299.1278	299.1283	1.7	281, 187, 165	*CTA, DGF, KWF

162	Sesamin	$C_{20}H_{18}O_6$	7.759	[M-H]-	354.1103	353.103	353.1020	-2.8	338, 163	*CTA, DGF
163	Schisandrin C	$C_{22}H_{24}O_6$	10.167	[M+H]+	384.1573	385.1646	385.1652	1.6	370, 315, 300	*CTA, LMN, AVO, PAP
164	Arctigenin	$C_{21}H_{24}O_6$	29.065	**[M-H]-	372.1573	371.15	371.1509	2.4	356, 312, 295	AVO
165	7-Oxomatairesinol	$C_{20}H_{20}O_7$	30.089	**[M+H]+	372.1209	373.1282	373.1297	4.0	358, 343, 328, 325	*LMN, ORN
166	Schisantherin A	$C_{30}H_{32}O_9$	37.579	[M+H]+	536.2046	537.2119	537.2115	-0.7	519, 415, 385, 371	*KWF, BNA, CTA, PER
167	Pinoresinol	$C_{20}H_{22}O_6$	40.958	**[M-H]-	358.1416	357.1343	357.1336	-2.0	342, 327, 313, 221	*GRF, AVO
168	7-Hydroxymatairesinol	$C_{20}H_{22}O_7$	47.587	[M-H]-	374.1366	373.1293	373.1283	-2.7	343, 313, 298, 285	*APL, NEC
169	Secoisolariciresinol-sesquilignan	$C_{30}H_{38}O_{10}$	59.607	[M-H]-	558.2465	557.2392	557.2387	-0.9	539, 521, 509, 361	*AVO, CTA
170	Schisandrol B	$C_{23}H_{28}O_7$	63.253	[M+H]+	416.1835	417.1908	417.1929	5.0	224, 193, 165	AVO
171	Schisandrin B	$C_{23}H_{28}O_6$	81.572	[M+H]+	400.1886	401.1959	401.1949	-2.5	386	CTA
**Stilbenes**										
172	Piceatannol 3-*O*-glucoside	$C_{20}H_{22}O_9$	8.335	[M-H]-	406.1264	405.1191	405.1172	-4.6	243	*CTA, AVO
173	Resveratrol	$C_{14}H_{12}O_3$	31.317	**[M-H]-	228.0786	227.0713	227.0709	-1.8	212, 185, 157, 143	*CTA, AVO, DGF
174	Resveratrol 5-*O*-glucoside	$C_{20}H_{22}O_8$	38.063	**[M-H]-	390.1315	389.1242	389.1245	0.8	227	*PSN, POM, KWF
175	3'-Hydroxy-3,4,5,4'-tetramethoxystilbene	$C_{17}H_{18}O_5$	43.904	[M+H]+	302.1154	303.1227	303.1221	-2.0	229, 201, 187, 175	DGF
176	4-Hydroxy-3,5,4'-trimethoxystilbene	$C_{17}H_{18}O_4$	63.286	[M+H]+	286.1205	287.1278	287.1280	0.7	271, 241, 225	*CTA, DGF

*Compound was detected in more than one fruit peel samples, data presented in this table are from asterisk sample. **Compounds were detected in both negative [M-H]- and positive [M+H]+ mode of ionization while only single mode data was presented. Fruit peel samples were mentioned in abbreviations. Apple peel "APL", Apricot peel "APR", Avocado peel "AVO", Banana peel "BNA", Custard apple peel "CTA", Dragon fruit peel "DGF", Grapefruit peel "GRF", kiwifruit peel "KWF", Lime peel "LMN", Mango peel "MNG", Melon peel "MEL", Nectarine peel "NEC", Orange peel "ORN", Papaya peel "PAP", Passionfruit peel "PSN", Peach peel "PEC", Pear peel "PER", Pineapple peel "PIN", Plum peel "PLM" and Pomegranate peel "POM"

(Apple Peel)

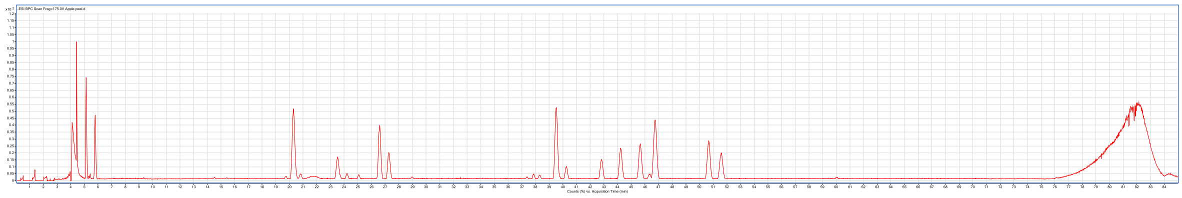

(Apricot peel)

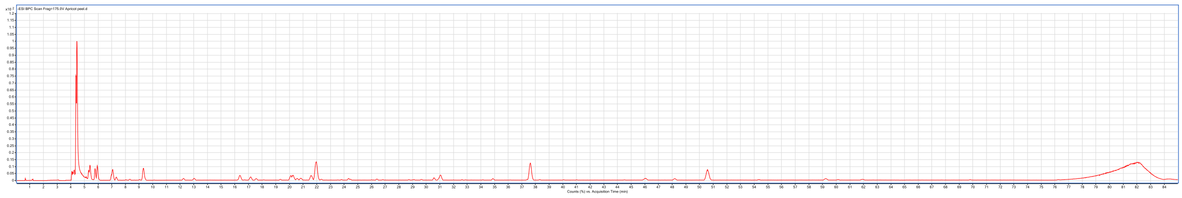

(Avocado peel)

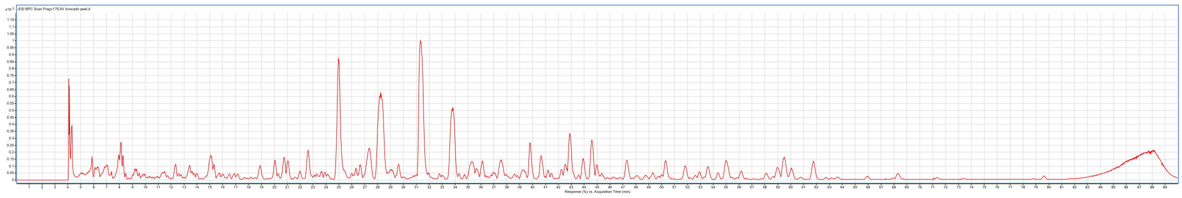

(Banana peel)

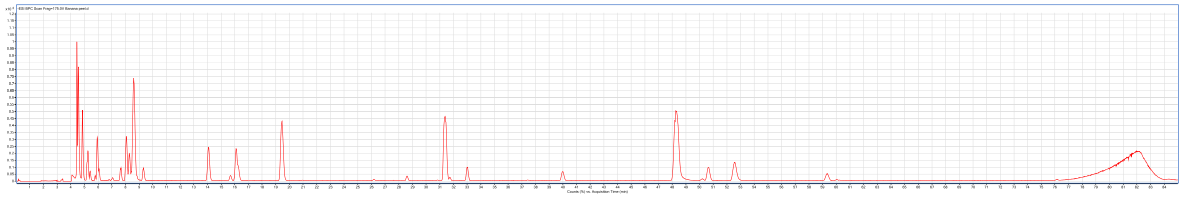

(Custard apple peel)

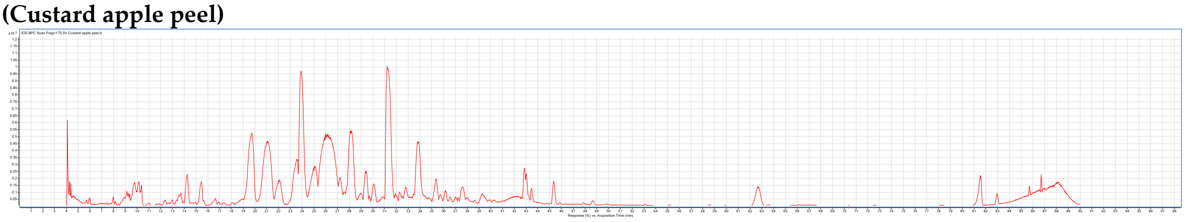

(Dragon fruit peel)

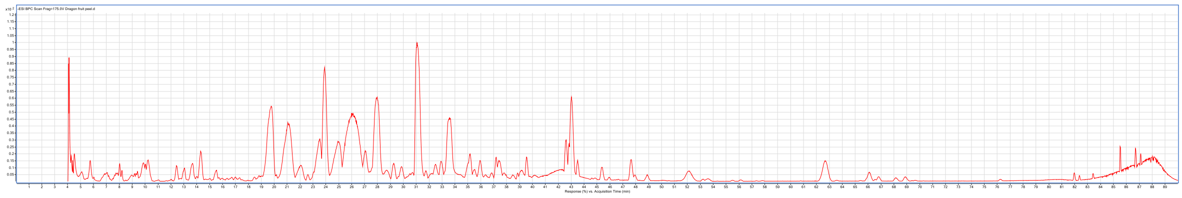

(Grapefruit peel)

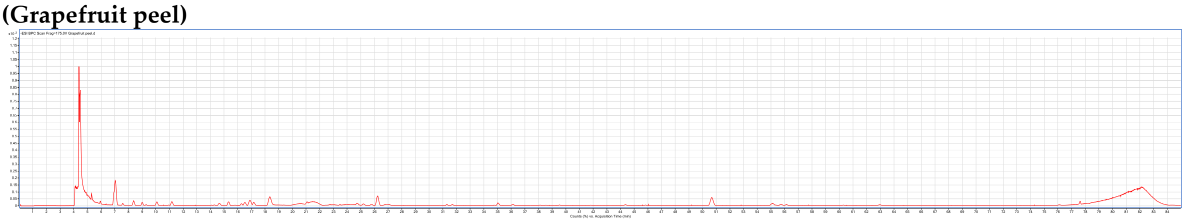

(Kiwifruit peel)

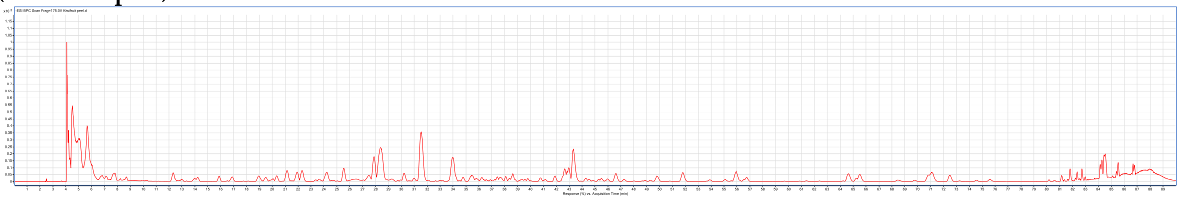

(Lime peel)

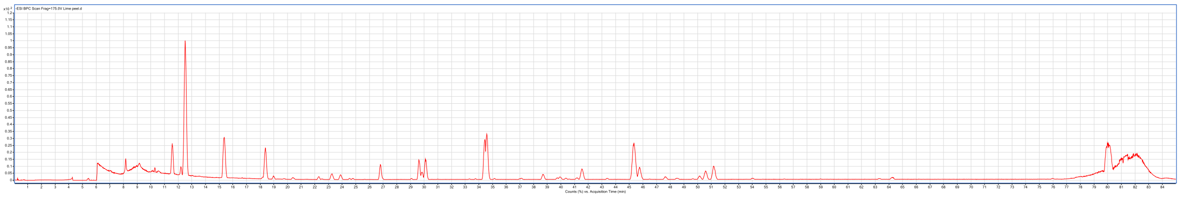

(Mango peel)

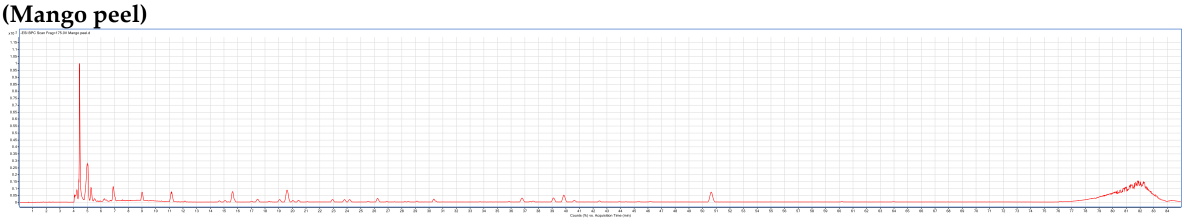

(Melon peel)

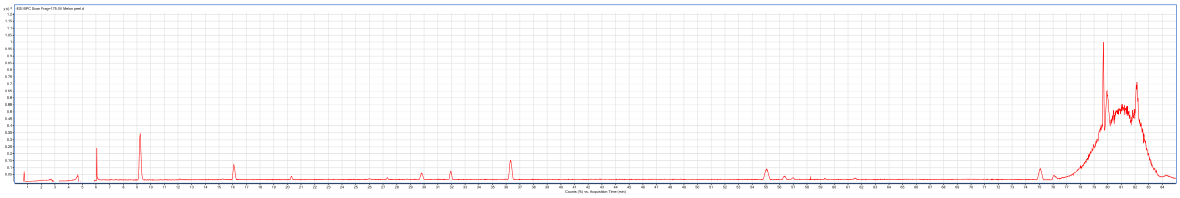

(Nectarine peel)

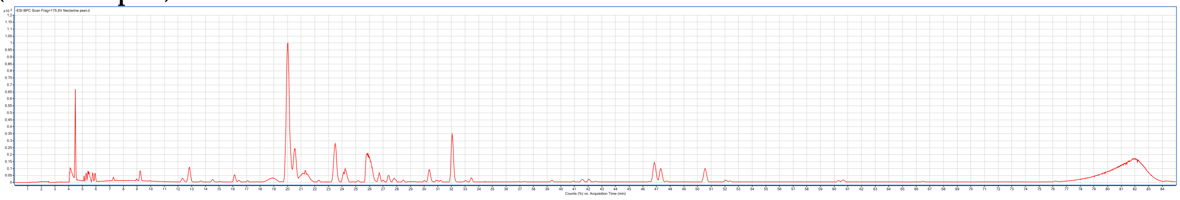

(Orange peel)

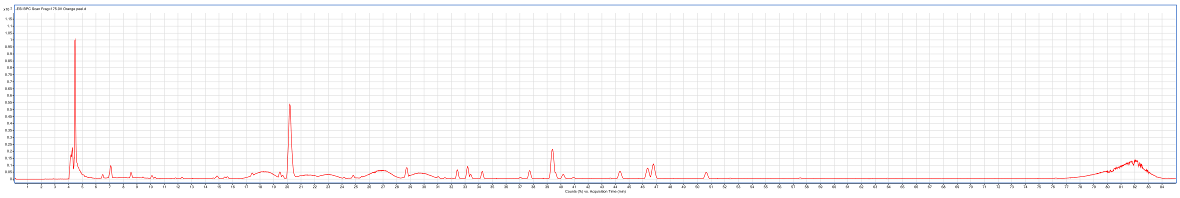

(Papaya peel)

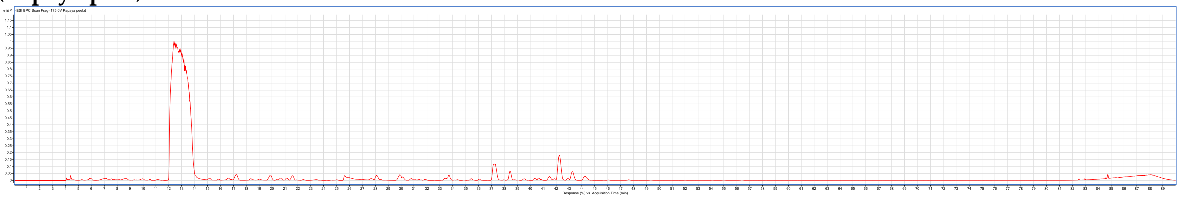

(Passionfruit peel)

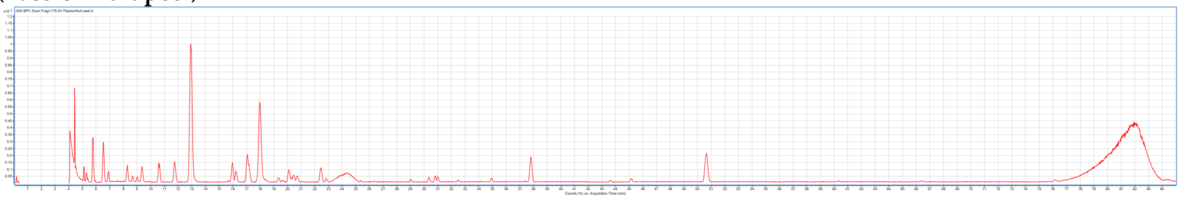

(Peach peel)

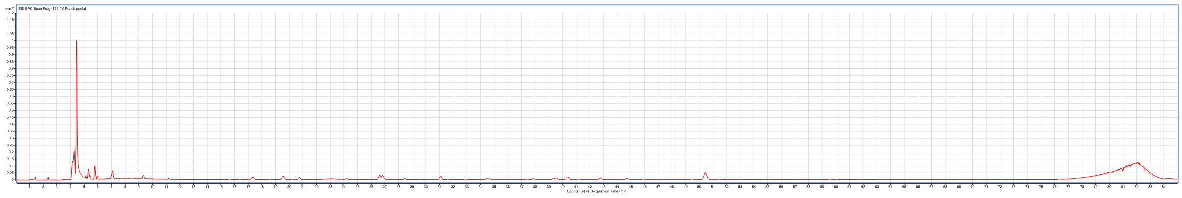

(Pear peel)

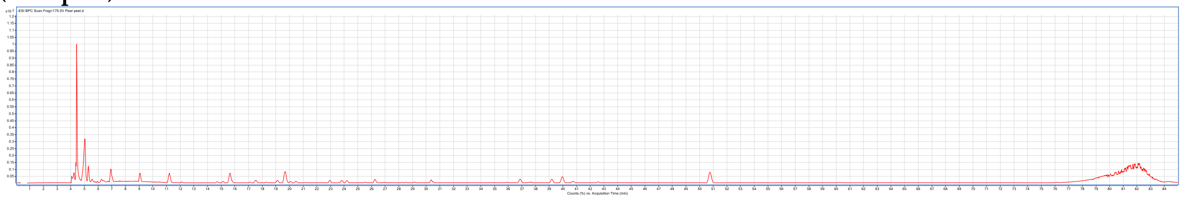

(Pineapple peel)

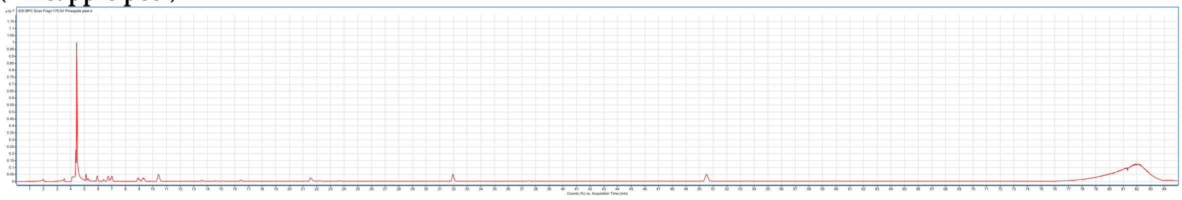

(Plum peel)

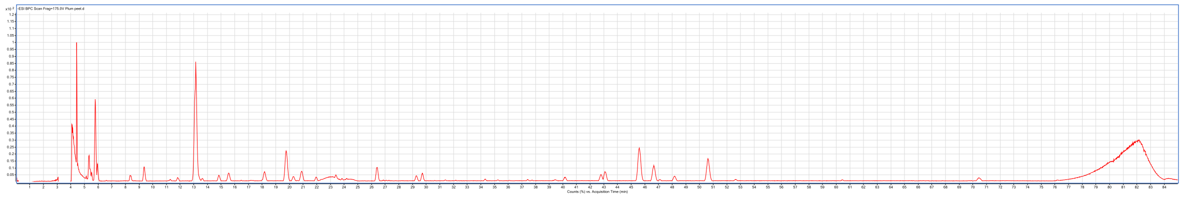

(Pomegranate peel)

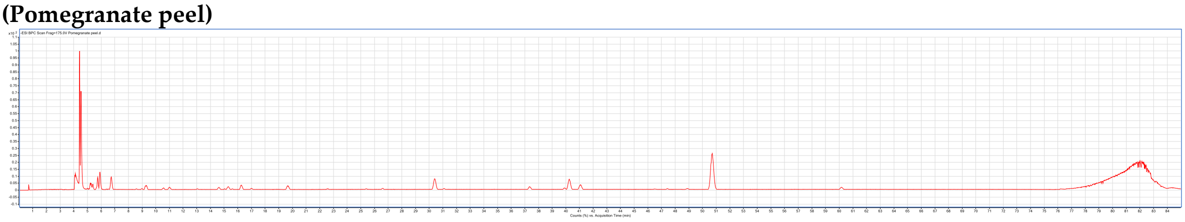

Figure S1: Characterization of phenolic compounds in different fruit peels by LC-ESI-QTOF-MS/MS. Base peak chromatogram (BPC) of twenty fruit peel samples in negative mode of ionization.

(Apple Peel)

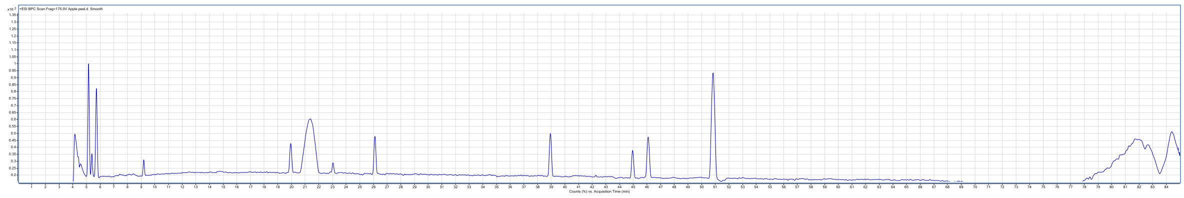

(Apricot peel)

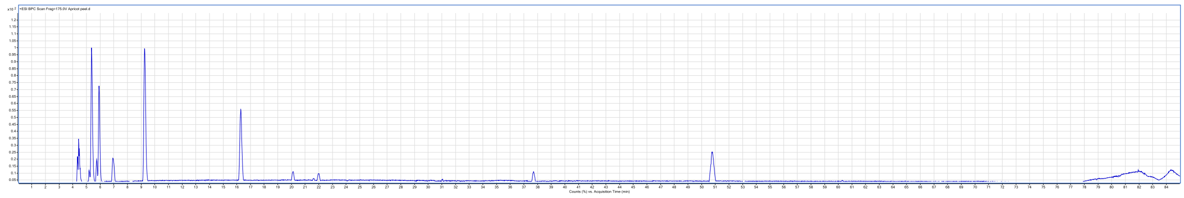

(Avocado peel)

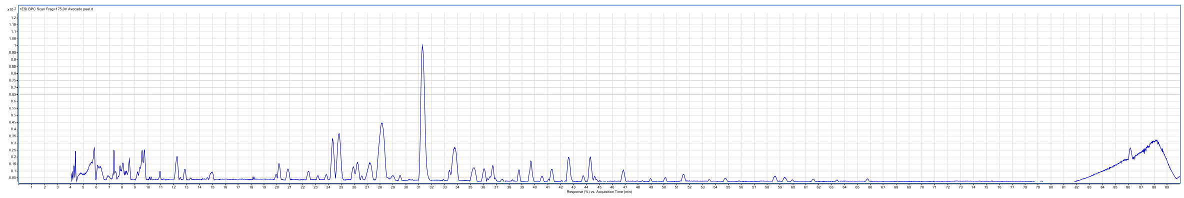

(Banana peel)

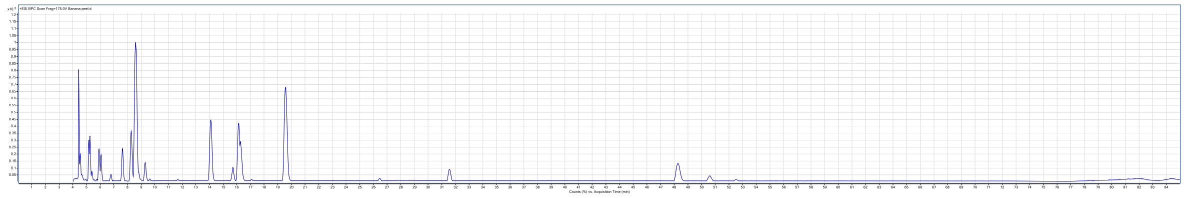

(Custard apple peel)

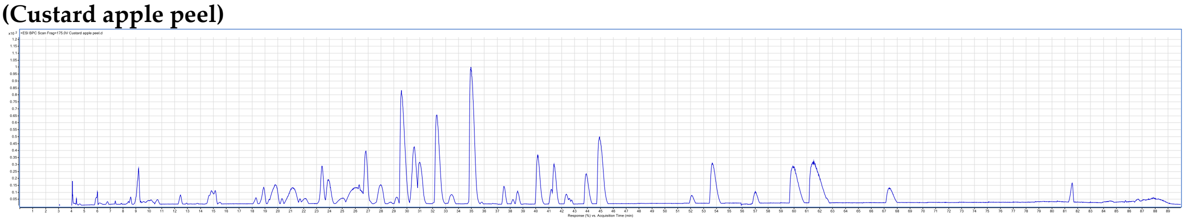

(Dragon fruit peel)

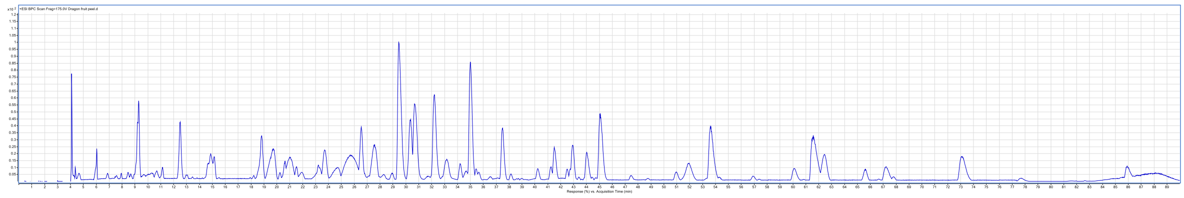

(Grapefruit peel)

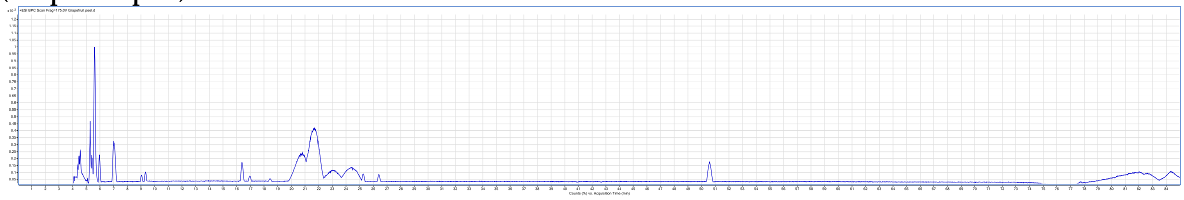

(Kiwifruit peel)

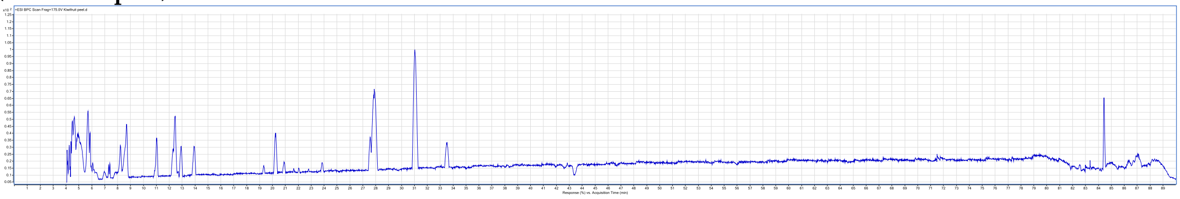

(Lime peel)

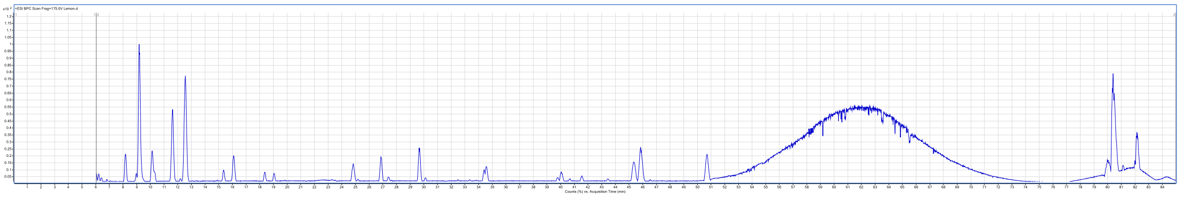

(Mango peel)

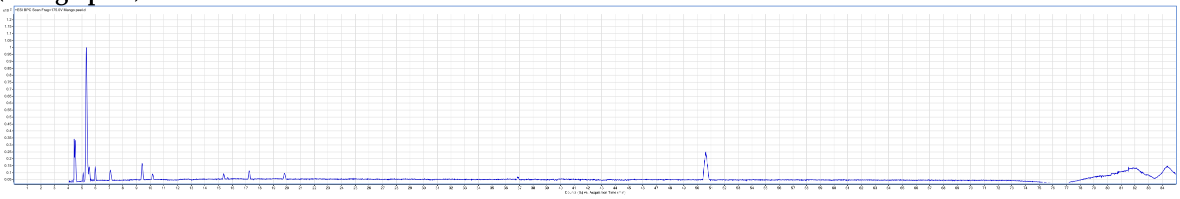

(Melon peel)

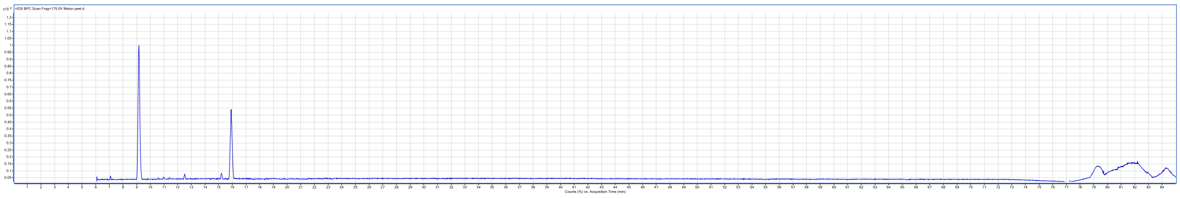

(Nectarine peel)

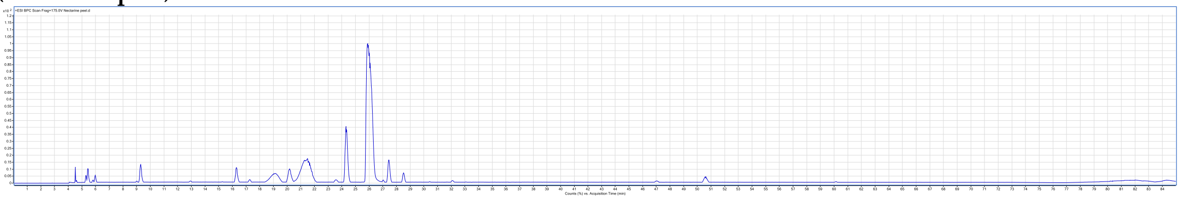

(Orange peel)

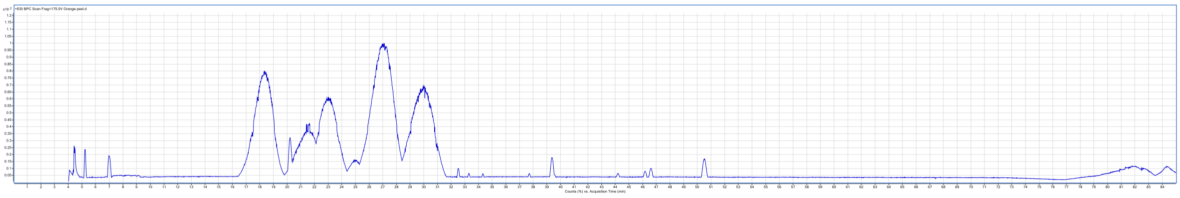

(Papaya peel)

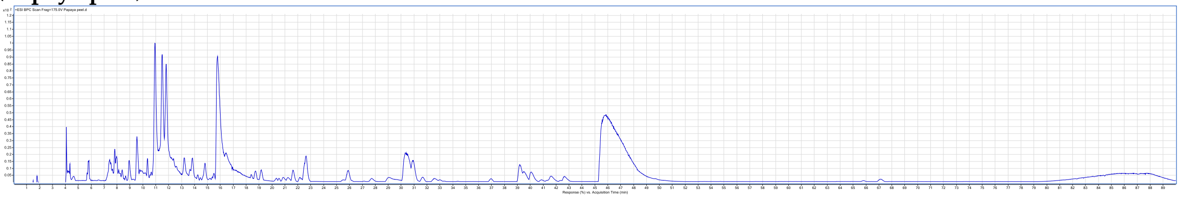

(Passionfruit peel)

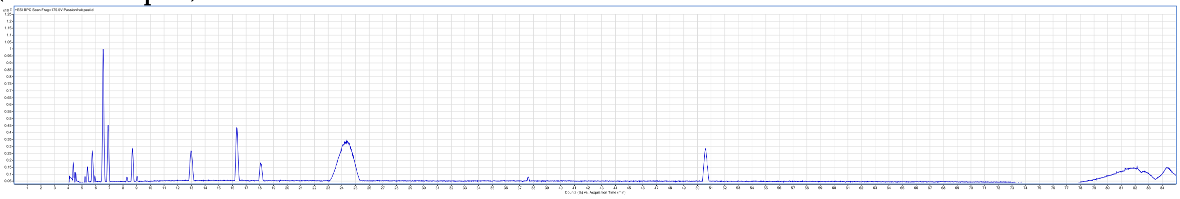

(Peach peel)

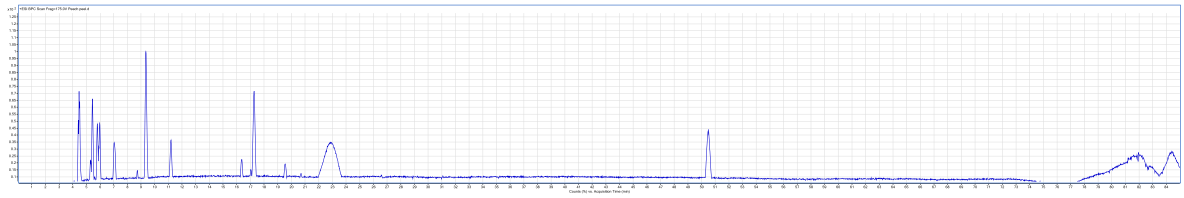

(Pear peel)

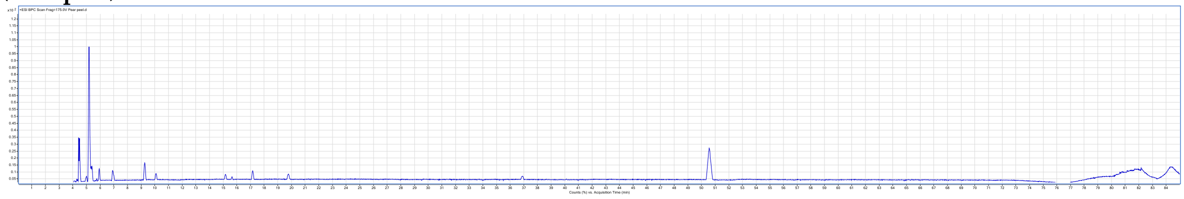

(Pineapple peel)

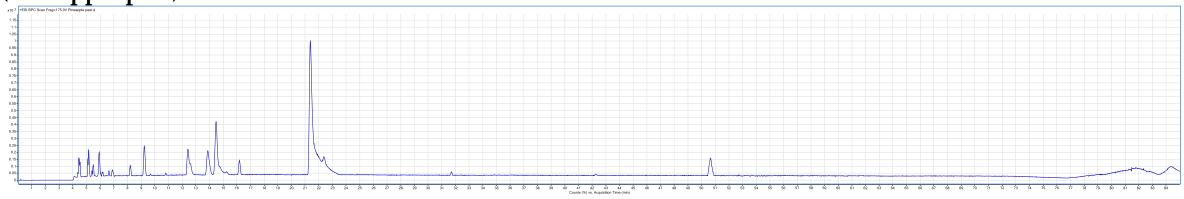

(Plum peel)

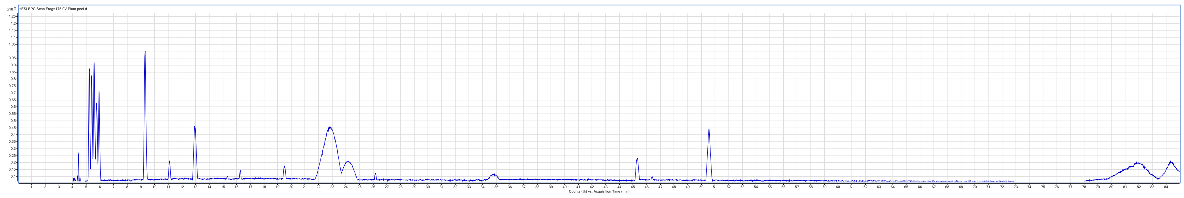

(Pomegranate peel)

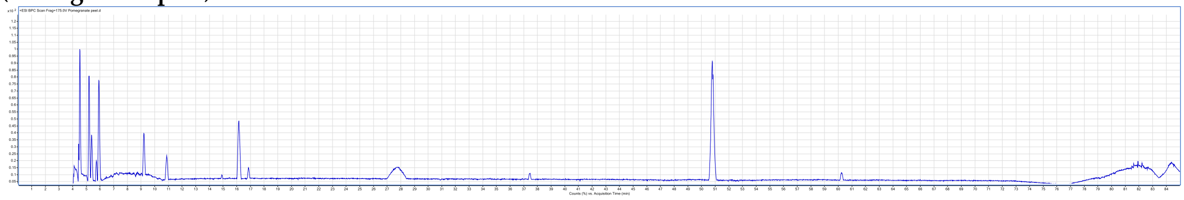

Figure S2: Characterization of phenolic compounds in different fruit peels by LC-ESI-QTOF-MS/MS. Base peak chromatogram of twenty fruit peel samples in positive mode of ionization.
